# Modules of co-occurrence in the cyanobacterial pan-genome reveal functional associations between groups of ortholog genes

**DOI:** 10.1371/journal.pgen.1007239

**Published:** 2018-03-09

**Authors:** Christian Beck, Henning Knoop, Ralf Steuer

**Affiliations:** Humboldt-Universität zu Berlin, Institut für Theoretische Biologie (ITB), Berlin, Germany; Universidad de Sevilla, SPAIN

## Abstract

Cyanobacteria are a monophyletic phylogenetic group of global importance and have received considerable attention as potential host organisms for the renewable synthesis of chemical bulk products from atmospheric CO_2_. The cyanobacterial phylum exhibits enormous metabolic diversity with respect to morphology, lifestyle and habitat. As yet, however, research has mostly focused on few model strains and cyanobacterial diversity is insufficiently understood. In this respect, the increasing availability of fully sequenced bacterial genomes opens new and unprecedented opportunities to investigate the genetic inventory of organisms in the context of their pan-genome. Here, we seek understand cyanobacterial diversity using a comparative genome analysis of 77 fully sequenced and assembled cyanobacterial genomes. We use phylogenetic profiling to analyze the co-occurrence of clusters of likely ortholog genes (CLOGs) and reveal novel functional associations between CLOGs that are not captured by co-localization of genes. Going beyond pair-wise co-occurrences, we propose a network approach that allows us to identify modules of co-occurring CLOGs. The extracted modules exhibit a high degree of functional coherence and reveal known as well as previously unknown functional associations. We argue that the high functional coherence observed for the modules is a consequence of the similar-yet-diverse nature of cyanobacteria. Our approach highlights the importance of a multi-strain analysis to understand gene functions and environmental adaptations, with implications beyond the cyanobacterial phylum. The analysis is augmented with a simple toolbox that facilitates further analysis to investigate the co-occurrence neighborhood of specific CLOGs of interest.

## Author summary

Cyanobacteria are photoautotrophic prokaryotes of global importance and offer great potential as host organisms for the renewable synthesis of chemical bulk products, including biofuels, from atmospheric CO_2_. As yet, however, research has mostly focussed on a small number of model strains and the genetic inventory of the cyanobacterial phylum is still insufficiently understood. The rapidly increasing availability of fully sequenced cyanobacterial genomes opens new and unprecendented possibilities to study the diversity of cyanobacterial strain in the context of the cyanobacterial pan-genome. Here, we seek to understand the genetic inventory of individual cyanobacterial strains based on the hypothesis that genes that are functionally related also co-occur within the genomes of different strains. We confirm this hypothesis by in depth analysis of co-occurrence that goes beyond pair-wise co-occurrences. We show that co-occurrence does not imply co-localization on the genome. Our work provides a novel approach to infer gene function and highlights the importance of a multi-strain analysis, with implications beyond the analysis of the cyanobacterial phylum.

## Introduction

Cyanobacteria are photosynthetic prokaryotes of global importance and recently gained renewed interest as a resource for natural products [1, 2] and as host organisms for the synthesis of renewable bulk chemicals [3]. Cyanobacteria exhibit highly diverse morphologies and are known to inhabit diverse environments, including lakes, oceans, arctic rocks, desert crusts, hot springs, and rice fields. In addition to the use of oxygenic photosynthesis as a primary source of reducing power and energy, many cyanobacteria are capable to assimilate atmospheric nitrogen, making cyanobacteria key players in the global nitrogen cycle. Despite their ecological and biotechnological importance, however, many aspects of cyanobacterial diversity are still insufficiently understood. In this respect, the increasing availability of fully sequenced cyanobacterial genomes [4, 5] opens unprecedented opportunities to delineate cyanobacterial diversity and the physiological adaptations from a genomic perspective.

Comparative genome analysis, in particular the analysis of the bacterial pan-genome, is established for more than a decade [6, 7]. Early applications include a comparison of 8 Streptococcus genomes [8], followed by an analysis of 17 *Escherichia coli* genomes [9], and similar studies for *Legionella pneumophila* [10], *Haemophilus influenzae* [11], and twelve closely related strains of the cyanobacterial genus Prochlorococcus [12]. Later studies considered an increasing number of strains [13, 14], the relationship between conserved genes and gene essentiality [15], as well as an analysis of niche-specific differences [16]. Several toolboxes for comparative genome analysis and the identification of ortholog genes have been described in the literature [17–21].

Here, we seek to obtain a better understanding of cyanobacterial diversity and the genetic inventory of strains. To this end, we conduct a comparative analysis of 77 fully sequenced and assembled cyanobacterial genomes. Beyond the analysis of the pan- and core-genome, we are specifically interested in the co-occurrence of clusters of likely ortholog genes (CLOGs). We hypothesize that genes with related functions also co-occur within genomes that constitute the pan-genome. As yet, however, co-occurrence has been primarily considered in the context of co-localization. Typical examples where co-occurrence and co-localization coincide are operons, sets of genes that are under control of a single promotor and act as a functional unit [22]. More general, however, sets of genes that constitute a functional unit must not necessarily be co-localized or be under the control of a single promotor. In the following, we therefore distinguish between co-localization (close proximity of genes on a genome) and co-occurence (genes that occur within a genome if and only if another gene is present).

To account for such more general functional relationships, Pellegrini et al. [23] introduced the concept of phylogenetic profiles. The phylogenetic profile of a CLOG is defined as an *N*-dimensional string that describes in which genomes of a set of *N* organisms the respective ortholog gene is present. CLOGs with similar phylogenetic profiles co-occur in different organisms—a fact that is indicative of a putative functional relationships between the respective CLOGs [23]. Phylogenetic profile comparisons have been applied to analyze genome architecture and to predict protein function [24–27]. While pair-wise co-occurrences can be straightforwardly detected, the identification of larger functional units, however, is not straightforward and involves the analysis of the community structure of large networks—a computationally nontrivial task. Here, we propose a network approach to extract co-occurring functional units from the pan-genome: we consider CLOGs as nodes in a network that are connected by (weighted) links if they co-occur within cyanobacterial strains. Based on a community-detection algorithm we then identify functional units of CLOGs, denoted as modules of co-occurring CLOGs, and provide an in-depth discussion of putative functional relationships. We argue that the high degree of functional coherence observed for the identified modules is a consequence of the similar-yet-diverse nature of the cyanobacterial phylum, with implications beyond the analysis of cyanobacteria.

The paper is organized as follows. In the first two sections, we briefly summarize several key properties of the cyanobacterial pan- and core genome. In the third section, we computationally identify co-occurring CLOGs within the cyanobacterial pan-genome. In the following section, we extend the analysis beyond pair-wise relationships and introduce the weighted co-occurrence network that is subsequently used to identify modules of co-occurring CLOGs. In the fifth section, we show that co-occurrence does not imply co-localization. Within the final sections we discuss examples of modules of co-occurring CLOGs and demonstrate that the identified modules indeed correspond to functional associations and provide novel hypotheses for gene function. To facilitate further analysis, the manuscript is supplemented with the software toolbox *SimilarityViewer* to allow for the exploration of co-occurrences beyond the selected examples discussed here.

## Results

### The cyanobacterial pan-genome revisited

Starting point of our analysis are 77 sequenced cyanobacteria sourced from the NCBI GenBank database. To avoid bias due to incomplete genomes, only completely assembled chromosomes, together with their associated plasmids (132 plasmids total) were selected. For later reference, the strain *Escherichia coli* O111:H (denoted as *E. coli* in the following) was included within the analysis. Orthology of all identified genes was determined based on an all-against-all BLASTp search as described previously [14]. Gene pairs with a high BLASTp score and bidirectional hit rate (BHR) were grouped together and subsequently clustered into cluster of likely ortholog genes, denoted as CLOGs. See section ‘Materials and methods’ for computational details. Due to their unique properties, the *Cyanobacterium* UCYN-A, an endosymbiont with a highly reduced genome [28], and *E. coli* were not part of the initial core- and pan-genome analysis. Their CLOGs were kept for later analysis.

**Fig 1:**
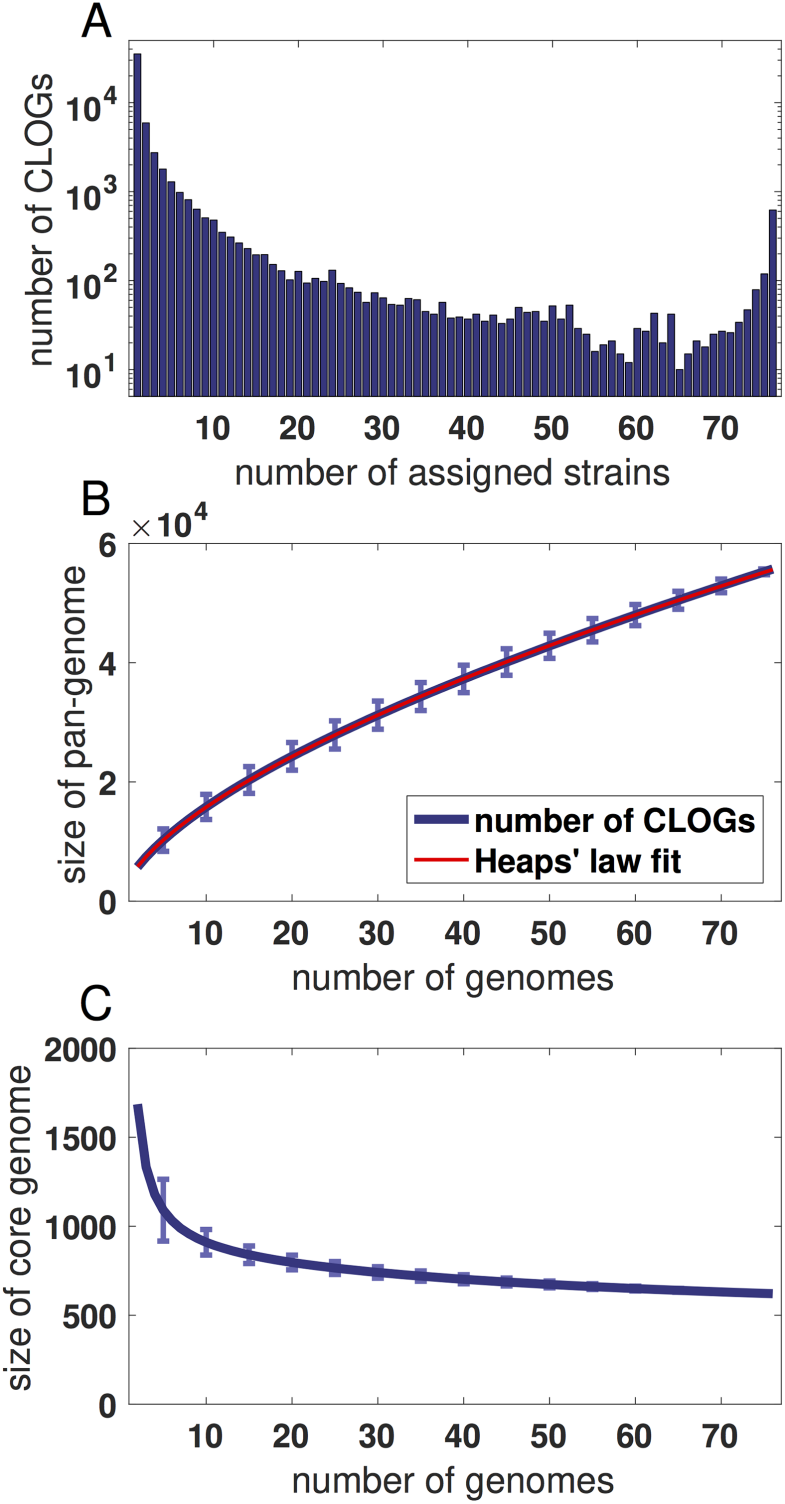
The cyanobacterial core and pan-genome. (A) The distribution of CLOGs as a function of the number of assigned strains. (B) The size of the pan-genome estimated for an increasing number of strains. The blue line indicates the mean size of the pan-genome, error bars indicate the standard deviation of 10^4^ randomly sampled subsets of strains. The red line shows a least squares fit of the power law *p* ∼ *N*^*g*^ (Heaps’ law), with *p* denoting the size of pan-genome and *N* the number of genomes. The estimated exponent *g* = 0.62 indicates an open pan-genome. (C) The size of the cyanobacterial core-genome estimated for an increasing number of strains. The blue line indicates the mean size of the core-genome whereas error bars indicate the standard deviation of 10^4^ randomly sampled subsets of strains. The estimates of pan- and core-genome do not include genomes of *E. coli* and *Cyanobacterium* UCYN-A.

We distinguish between core CLOGs, present in all remaining 76 cyanobacterial strains, shared CLOGs, present in one or more but not in all strains, and unique CLOGs, identified only in a single strain. Overall, we identified a total of 58740 CLOGs consisting of 621 core CLOGs, 20005 shared CLOGs, and 38114 unique CLOGs. Strains with larger genomes tend to be associated with more shared CLOGs. In contrast, the number of unique CLOGs associated with a single strain depends also on the phylogenetic distance to its nearest neighbors—and is therefore biased by the coverage of the cyanobacterial phylum. The number of strains associated with each CLOG is shown in Fig 1A. The overall properties of the pan- and core-genome are in good quantitative agreement with previous studies, typically using a smaller number of strains [14, 16]. Core CLOGs constitute between 7.4% (*Acaryochloris marina* MBIC11017) and 33.5% (*Prochlorococcus marinus* str. MIT 9211) of all CLOGs in a given genome. Evaluating the pan-genome for a subset of strains allows us to extrapolate the expected increase in the size of the pan-genome for newly sequenced genomes. The data follows Heap’s law (Fig 1B), indicating approximately 450 genes with sub-threshold similarity to any known protein for each newly sequenced genome [29]. These numbers are in good agreement with the 21107 novel sub-threshold genes identified by recent de-novo sequencing of 54 cyanobacterial strains [4]. We note that extrapolation of the core genome (Fig 1C) should be interpreted with caution due to the inherent statistical caveats when estimating rare events from limited data [30].

### CLOG annotation and the cyanobacterial pan-metabolism

CLOGs can be assigned to functional categories based on the annotation of their constituent genes. As expected, annotation coverage in core CLOGs is high, with 589 of 621 (95%) of core CLOGs containing at least one gene with functional annotation (as sourced from GenBank, including unspecific annotation, such as ‘membrane protein’ but excluding annotations such as ‘hypothetical’ and ‘conserved hypothetical’). Consistent with other studies [14–16, 31], functional annotations of core CLOGs are enriched in categories related to cellular metabolism, transcription, translation, and DNA replication. As compared to core CLOGs, annotation coverage for shared and unique CLOGs is significantly lower with 44% (8,853 of 20,005) and 82% (31,132 of 38,114), respectively, annotated as hypothetical, predicted or unknown. As observed previously [14], annotation is often unspecific or varying in the exact wording, for example ‘photosystem II DII subunit’ and ‘photosystem II protein D2’. Yet we observe only few instances of conflicting annotations for two or more genes in one CLOG. While automated comparison of conflicting annotation is not straightforward, manual inspection of 1000 CLOGs comprised of at least two annotated genes revealed putative inconsistencies for only 2.5% of the CLOGs. That is, in less than three percent of cases with at least two semantically different annotations, the annotations could not be identified as coinciding at a first glance.

We are specifically interested in the cyanobacterial pan-metabolism. To this end, the constituent genes of each CLOG were matched against the KEGG (Kyoto Encyclopedia of Genes and Genomes) database [32] to identify, which CLOGs are associated to EC numbers. We obtained a total of 2361 metabolism-related CLOGs associated with a total of 2301 metabolic reactions. We note that enzymes (and hence CLOGs) may catalyze multiple reactions and multiple enzymes (and hence CLOGs) may catalyze the same reaction. Consistent with previous studies [14], core CLOGs are highly enriched in metabolic function, with 322 of 621 (51.9%) associated with one or more specific reaction. The ratio is significantly lower for shared and unique CLOGs, with only 1664 (8.3%) shared CLOGs and 408 unique CLOGs (1.1%) associated to one or more specific reactions. Nonetheless, due to the higher number of shared CLOGs, metabolic functionality is primarily encoded in the shared genome. Of the 2301 unique reactions that constitute the cyanobacterial pan-metabolism, 1839 reactions are associated with at least one shared CLOG.

### Co-occurring CLOGs indicate functional relationships

We seek to identify putative functional relationships between CLOGs based on the hypothesis that co-occurrence of CLOGs is indicative of a functional relationship. We first performed a right-tailed Fisher’s exact test to identify pairs of CLOGs who preferentially co-occur within the same strain (See section ‘Materials and methods’). Using the multiple test correction method for non-independent tests by Benjamini and Yekutieli [33], we obtained a critical p-value of 1.43 ⋅ 10^−6^ for an accepted false discovery rate (FDR) of FDR = 0.01. In consequence, we identified 581741 out of more than 1.7 ⋅ 10^9^ possible pairs of CLOGs whose occurrences are significantly correlated. We note that, by definition, co-occurrence only involves shared CLOGs. Although technically co-occurring, core CLOGs are not considered in the analysis. The full list of co-occurring CLOGs is provided in S1 Table.

Manual inspection of co-occurring CLOGs indeed points to functional relationships. For example, among the pairs with the lowest p-value are the two subunits of cytochrome bd plastoquinol oxidase (CLOGs 11458 and 11459), typically forming an operon. Another example is the co-occurrence between subunits of the hydrogenase maturation protein Hyp, specifically the co-occurence of HypA (CLOG 10002) and HypE (CLOG 12374), as well as of HypE and HypF (CLOG 11744). Importantly, these subunits are not in close proximity on the genome in about half of all strains (16 of 39). Likewise, genes that encode a tocopherol cyclase (EC 5.5.1.24, CLOG 9703) and a homogentisate phytyltransferase (EC 2.5.1.115, CLOG 10825) co-occur. Both CLOGs also co-occur with a CLOG encoding a 4-hydroxyphenylpyruvate dioxygenase (EC 1.13.11.27, CLOG 10837). The genes of this triplet are essential for the biosynthesis of Vitamin E but are not in close genomic proximity on any strain. In the following, we therefore focus on two distinct aspects of co-occurring CLOGs: Firstly, functional relationships must go beyond pairs and may involve groups of CLOGs. Secondly, the genes of co-occurring CLOGs must not necessarily be in close proximity on the genome. We therefore must distinguish between co-occurence within a genome and co-localization. Since co-localization of genes on genomes is already employed in functional analysis [34], we seek to investigate to what extend co-occurrence provides *additional* information that augments co-localization as an indicator of a functional relationship.

We note that, in addition to co-occurrence, also anti-occurrence can be studied. Mutually exclusive pairs of CLOGs, however, are typically associated with specific phylogenetic clades, such as an exclusive association to either *α*-cyanobacteria or *β*-cyanobacteria. In the following, we therefore focus on co-occurrence only. A brief analysis of anti-correlated pairs of CLOGs is provided in the supplement Fig A in S1 Text.

### Network analysis of co-occurring CLOGs

Pair-wise co-occurrences are not sufficient to fully reveal the underlying structure of functionally related CLOGs. We therefore seek to identify groups of CLOGs, denoted as modules, that co-occur across different genomes. To this end, we consider CLOGs as nodes in a network, such that two CLOGs are connected by a (weighted) link if their co-occurrence is statistically significant. We utilize a weight function *w*(*i*, *j*) between two co-occurring CLOGs *i* and *j* that is phylogeny-aware [26]. That is, links between CLOGs that co-occur in phylogenetically closely related genomes are assigned less weight than links between CLOGS that co-occur in phylogenetically distant genomes. In this way, the phylogenetic signal in the co-occurence of CLOGs is reduced. See section ‘Materials and methods’ for details.

**Fig 2:**
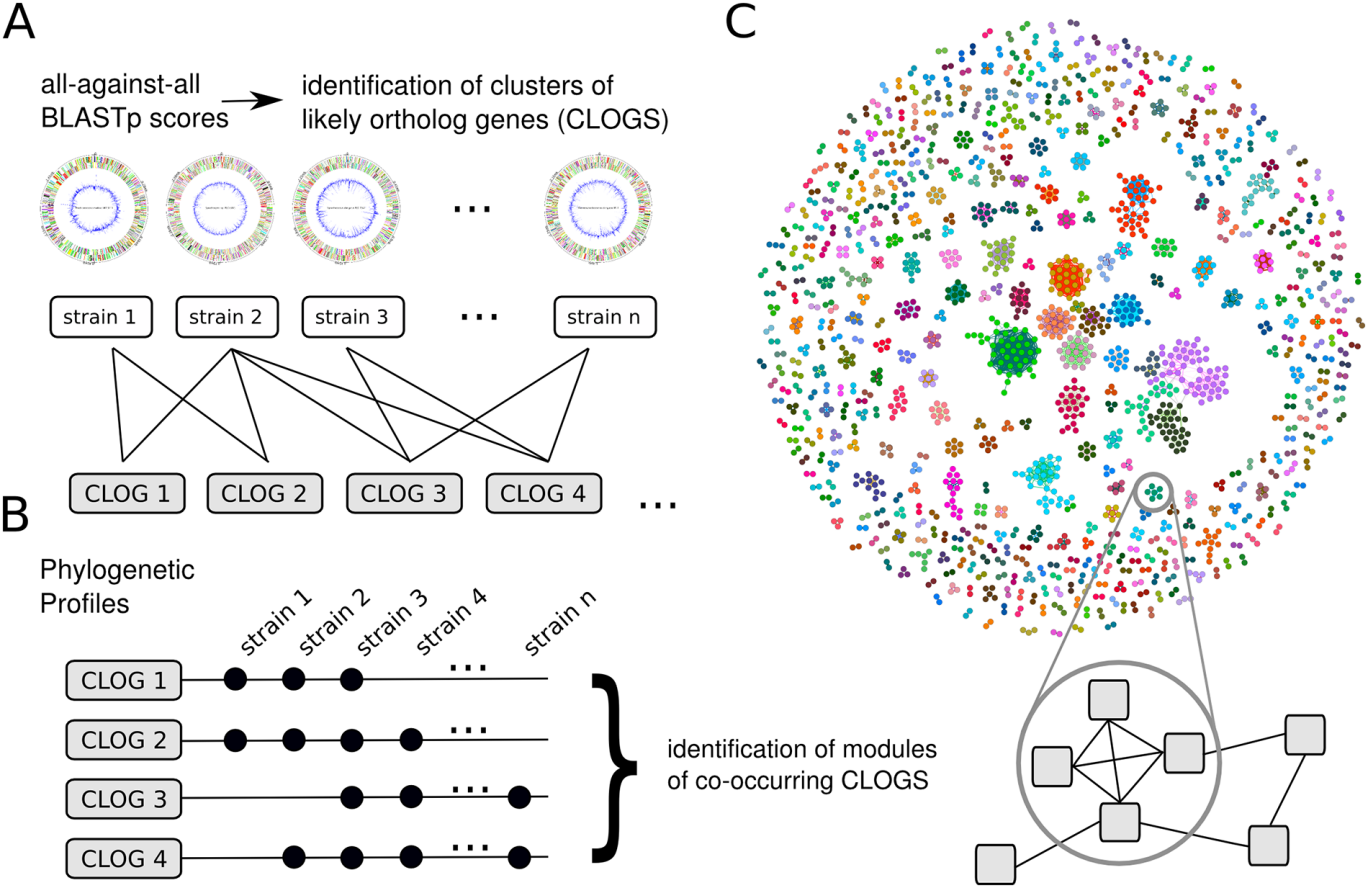
Network analysis of co-occurring CLOGs. (A) Orthologous genes are identified using an all-against-all BLASTp comparison and are grouped into cluster of likely orthologous genes (CLOGs). CLOGs are classified into three sets: core CLOGs (present in all strains), shared CLOGs (present in several but not all strains) and unique CLOGs (present in a single strain). (B) The phylogenetic profile of each CLOG indicates the set of strains whose genome is annotated with genes corresponding to the CLOG. Pair-wise co-occurrence of CLOGs is identified using the similarity of phylogenetic profiles. CLOGs are grouped into modules of co-occurring CLOGs using a community-detection algorithm. (C) A network view on co-occurring CLOGs. We identify a total of 563 modules with 1930 CLOGs. Circular genome maps were constructed using the CiVi tool [60].

Based on the resulting weighted co-occurrence network, modules of co-occurring CLOGs are identified using the algorithm of Blondel et al. [35]. The algorithm is based on heuristic modularity optimization, parameter-free and reasonably fast. We note that module identification is computationally hard and precise (non-heuristic) formulations are computationally intractable for large networks. Input to the algorithm is the weighted co-occurrence network using a threshold value of *w* = 0.65 as cutoff for minimal weight of edges. The results are highly robust with respect to different choices of the cutoff. The workflow is depicted in Fig 2.

The algorithm of Blondel results in 563 modules comprising a total of 1930 CLOGs. Most modules (542 of 563) are of size ten or less, 93 modules consist of three, 371 modules consist of only two CLOGs, respectively. All identified modules and their constituent CLOGs are listed in S2 Table. We note that, despite the correction for phylogenic proximity, large modules typically reflect different subgroups of cyanobacteria. For example, the largest module consists of 48 CLOGs with seemingly unrelated functional annotation who (co-)occur in most *β*-cyanobacteria, with the exception of both Gloeobacter strains. Similar, the second largest module (41 CLOGs of which 25 have no annotated function) is mostly associated with *α*-cyanobacteria, excluding all but two Prochlorococcus strains. Smaller modules, however, are typically not associated to particular clades or subtrees and indicate functional relationship between CLOGs.

### Co-occurrence and co-localization

Prior to an in-depth analysis of putative functional relationships between CLOGs, we evaluate to what extent modules of co-occurring CLOGs reflect co-localization on the genome. In particular, we seek to investigate whether modules of co-occurring CLOGs primarily recapitulate the (known) co-localization structure of functionally related CLOGs. To this end, we tested all modules for co-localization and operon-like structures: In brief, we estimate a (strain-specific) adjacency score AS that measures to what extent all genes within a module are located in close proximity on the genome of a specific strain. The strain-specific adjacency score of a module is AS = 1 if all genes corresponding to the module are separated by less than ten open reading frames from another gene of that module, and AS = 0 if no two genes within a module are located less than ten open reading frames from each other. The aAS of the module is then given as the average of the AS of all constituent strains of a module. See section ‘Materials and methods’ for details.

**Fig 3:**
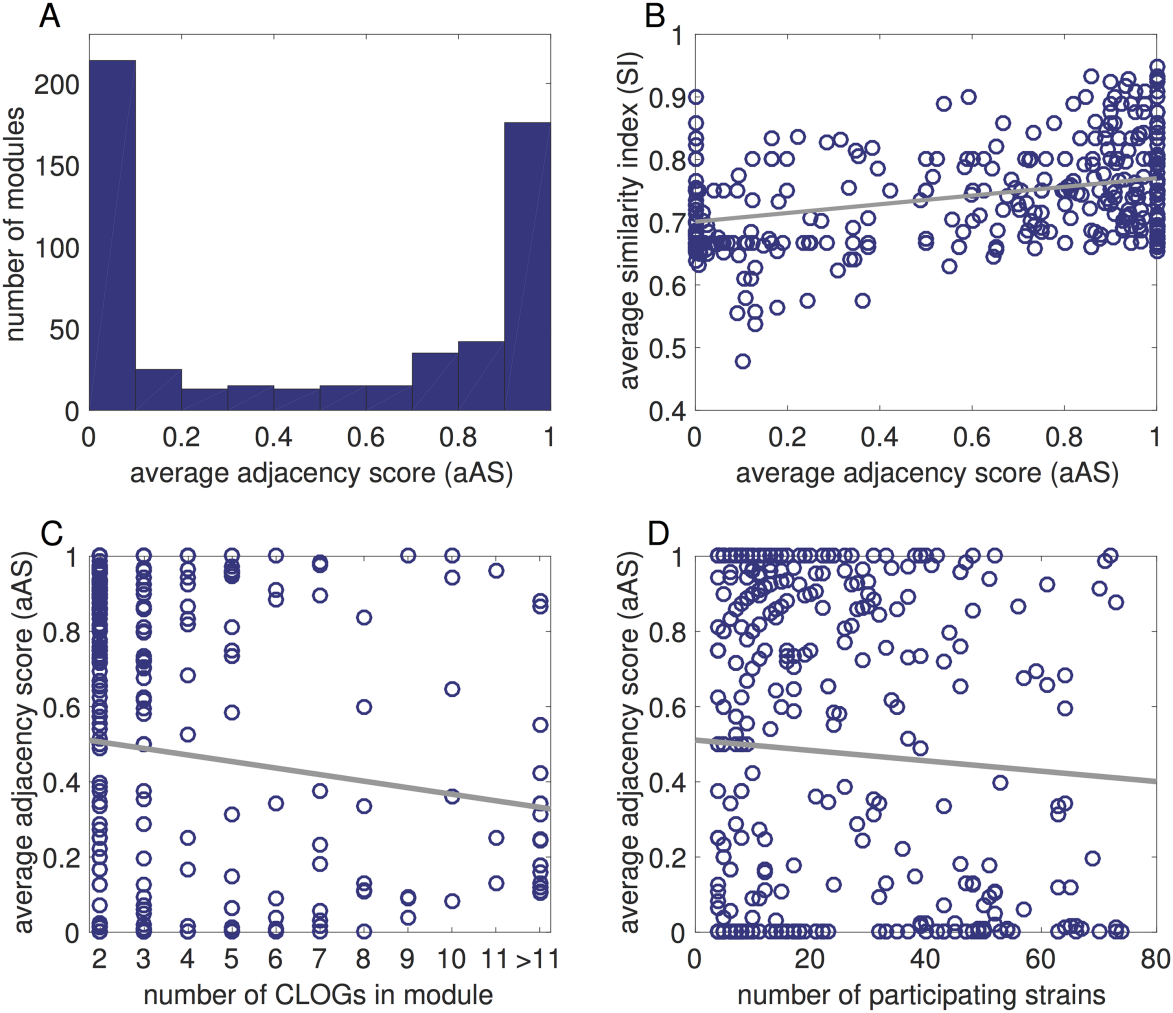
Genomic proximity of co-occurring CLOGs. The average adjacency score (aAS) measures the co-localization of CLOGs grouped into co-occurring modules. (A) A histogram of the average adjacency score (aAS). The histogram shows a clear dichotomy between modules whose constituent CLOGs (and hence genes) are co-localized in all genomes (aAS ≈ 1) and modules whose genes are not co-localized (aAS ≈ 0). (B) A scatter plot between the similarity score, measuring the quality of co-occurrence, and the aAS. The plot indicates that there is a positive but weak correlation between the genomic proximity of the genes comprising a module (represented by the aAS) and the quality of co-occurrence. The straight line corresponds to a linear regression and serves as a guide to the eye. (C) A scatterplot between the number of CLOGs associated to module and the aAS. While larger modules tend to have a lower aAS, the aAS scores are relatively well distributed with respect to the number of CLOGs in a module. (D) A scatterplot between the number of strains associated to a a module and the aAS. The number aAS is again relatively well distributed with respect to number of participating strains. In both plots the straight line indicates a linear regression and serves as a guide to the eye.

The distribution of the aAS for each module is shown in Fig 3A. We observe a dichotomy between modules whose constituent CLOGs (and hence genes) are co-localized in all genomes (aAS ≈ 1) and modules whose genes are not co-localized (aAS ≈ 0). The strict dichotomy is partly explained by the fact that a large number of modules (371 of 563) consist only of two CLOGs, hence the respective (strain-specific) AS can only be either zero or one. Interestingly, the quality of co-occurrences within a module, as measured by the average similarity index (the average similarity of the phylogenetic profiles of two CLOGs, see section ‘Materials and methods’), shows only a weak correlation with genomic adjacency (Fig 3B). Modules with a low average similarity index between its CLOGs may exhibit a similar range of adjacency scores as modules with high similarity index. We further tested the relationship between the aAS of a module and the number of participating CLOGs (Fig 3C) and the number of participating strains (Fig 3D).

In general, modules with high aAS often correspond to known operons. For example, module 9 (aAS = 0.87) contains 21 CLOGs related to the formation of nitrogenase (EC 1.18.6.1), whose genes are arranged in one to three operon-like groups in the genome of most strains. The operon structure of nitrogenase-related genes was previously described by Mulligan and Haselkorn [36]. But modules with lower aAS may also consist of CLOGs that functionally closely related. For example module 293 (aAS = 0) consists of two CLOGs that are annotated as a substrate-binding and membrane subunit of a carbohydrate ABC transporter. The co-occurring subunits are not in close proximity in any of respective cyanobacterial genomes.

We note that the genomic proximity of genes is rather conserved in general, with 317 of 563 modules having the same AS in all associated strains, but it can also vary drastically between strains. For example module 52 (aAS = 0.34) consists of six genes for nitrate reductase (EC 1.7.7.2) and five proteins associated with its assembly. Despite their close functional relationship, the corresponding genes are organized in operon-like structures in only 13 strains (mostly Synechococcus) but are spread across the genomes of 21 other strains. Variations of the genomic adjacency between strains are not straightforward. Neither smaller, streamlined genomes, nor strains with genes organized in multiple plasmids feature a generally difference in the number of operon-like structures (see Fig B and C in S1 Text).

In summary, we conclude that modules of co-occurring genes do not merely recapitulate co-localization and that analysis of the genomic neighborhood and co-occurrence analysis supplement each other to determine candidates for functionally related genes.

### Modules of co-occurring CLOGs indicate functional relationships

**Fig 4:**
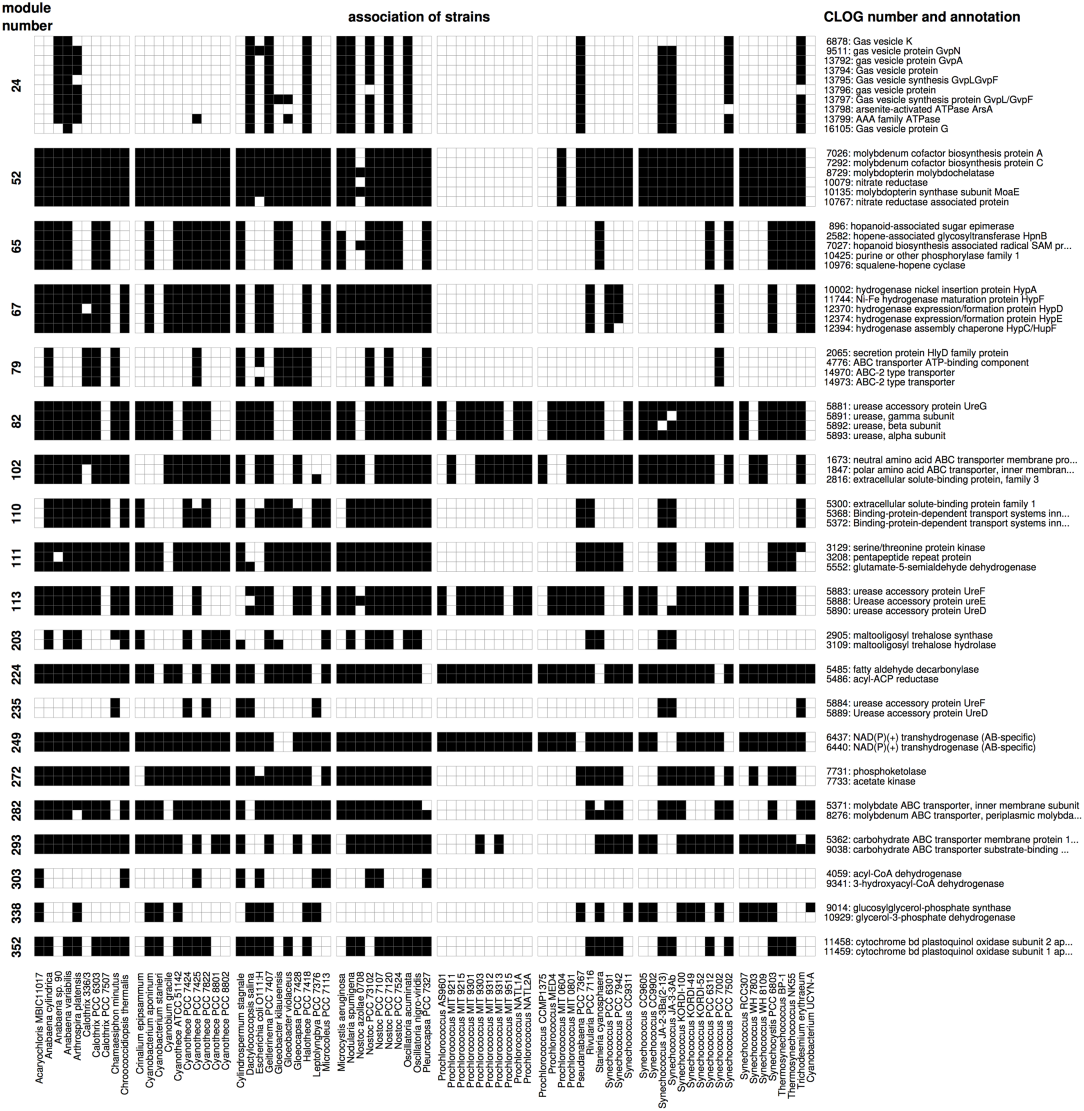
Selected modules of co-occurring CLOGSs and their associated strains. A black box indicates if a CLOG (y-axis) is associated with a specific strain (x-axis). The first column indicates the module number, the last column indicates the primary annotation of the respective CLOG. Shown is an excerpt of modules of co-occurring CLOGs.

To evaluate to what extent modules of co-occurrence provide novel hypotheses for putative functional relationships between CLOGs, we discuss 20 typical modules. The relationship between the selected modules and their constituent CLOGs are depicted in Fig 4. The full list of modules are provided in S2 Table.

The most straightforward instances of functional relationships between CLOGs are subunits of heteromultimeric proteins that co-occur across diverse genomes. For example, **module 249** (2 CLOGs, aAS = 0.99) consists of two CLOGs coding for the alpha and beta subunit of a NAD(P)+ transhydrogenase (EC 1.6.1.2) and **module 352** (2 CLOGs, aAS = 1) consists of the subunits I and II of cytochrome bd quinol oxidase (EC 1.10.3.14) [37]. **Module 67** (5 CLOGs, aAS = 0.73) consists of the NiFe-type hydrogenase maturation protein subunits HypA, HypC, HypD, HypE and HybF [38]. Interestingly, the sixth subunit HypB (CLOG 5882) is not present in this module. The subunit can often be found in multiple copies and is also present in cyanobacteria that do not harbor the other 5 subunits (e.g. *Leptolyngbya* PCC 7376, *Synechococcus* CC 9605, among others), suggesting a possible second function. **Module 82** (4 CLOGs, aAS = 0.68) consists of the alpha, beta, and gamma subunits of urease (EC 3.5.1.5) as well as the urease accessory protein UreG. Urease catalyzes the hydrolysis of urea into carbon dioxide and ammonia as a source of nitrogen. The protein complex assembly is assisted by the four chaperons UreD, UreE, UreF, and UreG [39, 40]. For most strains, the accessory proteins UreD, UreE, and UreF are grouped in **module 113** (3 CLOGS, aAS = 0.68). The remaining cyanobacteria that possess urease (e.g. *Cyanothece* PCC 7424, *Leptolyngbya* PCC 7376, *Trichodesmium* IMS101) have a modified UreD and UreF (**module 235**, 2 CLOGS, aAS = 0.78) but lack the UreE chaperon.

A second class of functional relationships are modules whose constituent CLOGs encode transporters. In cyanobacteria and other gram-negative bacteria, ABC (ATP binding cassette) transporters are usually comprised of 3 different molecular components: an ATP-binding/ hydrolyzing protein (NBD—nucleotide binding domain), one or two transmembrane proteins (TMD) building a homo- or heterodimeric structure, and a soluble, secreted substrate-binding protein (BP). In varying compositions they form a membrane spanning structure that can actively change its conformation to facilitate the transport of various compounds through the membrane [41, 42]. **Module 79** (4 CLOGs, aAS = 0.87) consists of two transmembrane proteins, one ATP-binding protein, and one soluble substrate binding protein comprising an ABC transporter with unclear specificity. **Module 102** (3 CLOGS, aAS = 0.87) groups CLOGs for one substrate-binding proteins as well as two transmembrane proteins for transport of neutral and charged amino acids respectively. These genes are typically found in an operon-like proximity on the genomes together with an ATP-binding protein. **Module 110** (3 CLOGs, aAS = 0.62) consists of two transmembrane proteins and one ATP-binding protein forming a putative polyamine transporter; **module 282** (2 CLOGs, aAS = 0.65) consists of a molybdate transporter that is assembled from a fused NBD-TMD protein as well as the substrate-binding protein. Interestingly, in 16 of the 47 strains these two genes are not in close proximity on the genome. **Module 293** (2 CLOGs, aAS = 0) consists of the transmembrane and the substrate-binding protein of a carbohydrate transporter. These CLOGs do not form an operon in any strain.

We note that ATP-binding NBD proteins are not always modularized with the corresponding transmembrane and substrate-binding proteins. It is known, that the ATP-binding proteins of different ABC transporters are highly conserved—up to a degree they can functionally substitute each other [41, 43]. The identity of different NBD-proteins can exceed 60% with a BLAST e-value of *e*^−100^ and less. The high degree of sequence similarity therefore results in multiple ATP-binding proteins being clustered together in a single CLOG (CLOGs 4775 and 4788), compromising the specific patterns of occurrence.

### Co-occurrences of CLOGs related to metabolic functions

A third class of functional relationships are modules whose constituent CLOGs encode proteins involved in a common metabolic pathway. For example, **module 272** (aAS = 0.4) consists of two CLOGs encoding for a phosphoketolase (EC 4.1.2.22) and an acetate kinase (EC 2.7.2.1), respectively. The phosphoketolase catalyzes the reaction of fructose 6-phosphate to erythrose 4-phosphate and acetyl phosphate, the latter is subsequently converted into acetate by the co-occurring acetate kinase. Therefore the module reflects a functional association, although both genes are not in close genomic proximity in 32 of 53 strains, including *Synechocystis* sp. PCC 6803. **Module 203** (aAS = 0.9) consists of two CLOGs that code for enzymes of the trehalose synthesis pathway, namely maltooligosyl trehalose synthase (EC 5.4.99.15) and maltooligosyl trehalose hydrolase (EC 3.2.1.141) [44]. **Module 65** (5 CLOGs, aAS = 0.31) is associated with the synthesis of hopanoids, which organize the lipid fraction of cell membranes [45]. The module consists of CLOGs whose genes code for the hopanoid-associated sugar epimerase (HpnA), hopene-associated glycosyltransferase (HpnB), squalene-hopene cyclase (HpnF), hopanoid biosynthesis associated radical SAM protein (HpnH) and a not further specified phosphorylase. All strains participating in this module also harbor at least one copy of the squalene synthase (EC 2.5.1.21), which exists in two variants and is therefore split into the CLOGs 10423 and 10424. **Module 224** (2 CLOGs, aAS = 0.91) consists of two CLOGs encoding an aldehyde decarbonylase [46] and an acyl-ACP reductase [47], respectively. The strict co-occurrence and operon-like structure of both genes has already been described in the context of cyanobacterial alkane biosynthesis [48]. **Module 303** (aAS = 0.9) consists of two CLOGs, acyl-CoA dehydrogenase (EC 1.3.8.7) and 3-hydroxyacyl-CoA dehydrogenase (EC 1.1.1.35)—both integral components of the degradation of fatty acids and branched-chain amino acid. Interestingly, in all cyanobacterial strains, genes encoding these enzymes form an operon-like structure around a gene that is either part of CLOG 19506 (no clear annotation) or CLOG 9342 (acetyl-CoA acetyltransferase, EC 2.3.1.16). The latter is part of the fatty acids degradation pathway, indicating a similar function of the genes in CLOG 16506. **Module 338** (2 CLOGS, aAS = 0.35) consists of the enzymes glucosylglycerol-phosphate synthase (EC 2.4.1.213) [49] and glycerol-3-phosphate dehydrogenase (EC 1.1.5.3), which are involved in the synthesis pathway of osmoprotective compound glucosylglycerol [50]. In (only) seven of the 23 strains harboring both CLOGs the corresponding genes are found in operon-like proximity. Recently, a gene with glycosylglycerol hydrolase activity was identified in Synechosystis sp. PCC 6803 [51]. The gene is not part of the module, as the respective CLOG is annotated in only 13 of the strains considered here, and hence does not strictly co-occur.

### Co-occurrences of CLOGs related to specific cellular functions

The final class of modules combines CLOGs related to specific cellular process. For example, **module 52** (6 CLOGS, aAS = 0.34) consists of CLOGs encoding molybdenum cofactor biosynthesis protein A and C, molybdopterin biosynthesis MoeA and MoeE proteins as well as a nitrate reductase and a nitrate reductase associated protein. The co-occurrence can be explained by the co-factor molybdopterin providing molybdenum to the reaction center of the nitrate reductase [52]. **Module 24** (8 CLOGs, aAS = 0.65) consists of 8 CLOGs related to the assembly of gas vesicles proteins. Gas vesicles allow cyanobacteria a controlled lateral movement in liquid medium. The module also contains CLOGs coding for two ATPases with unknown function that might be involved in vesicle formation or pumping processes. The genes of this module are found in close genomic proximity in 10 of the 16 participating genomes.

### Modules provide novel hypotheses for gene function

Of particular interest are modules that include CLOGs whose constituent genes are annotated with specific functions, as well as CLOGs whose constituent genes encode for unknown or putative regulatory proteins. Such modules may provide novel hypotheses about the functional role of genes with unknown function and provide additional insight into the organization of cellular processes. For example **module 111** (3 CLOGs, aAS = 0.07) consists of glutamate-5-semialdehyde dehydrogenase (EC 1.2.1.41) involved in the synthesis of essential amino acid L-proline as well as two CLOGs with likely regulatory functions, a pentapeptide repeat protein and a serine/threonine protein kinase. **Module 9** (21 CLOGs, aAS = 0.87) contains multiple CLOGs that are associated with the fixation of inorganic nitrogen, as well as five CLOGS corresponding to likely regulatory genes. In *Nostoc* sp. PCC 7120 these are asr1405 (hypothetical protein), all1432 (UBA/THIF-type binding protein, probable hesA), asl1434 (rop-like domain protein), all2512 (probable transcriptional regulator PatB), and asr2523 (TPR domain protein). The putative regulatory genes are located almost always in close genomic proximity to the other genes of module, suggesting a role of these genes in the process of nitrogen fixation.

Other modules involve only CLOGs of unknown function and therefore lack a straightforward functional interpretation. In this case, the shared traits of the strains in which the CLOGs co-occur may provide additional information. For example, **module 3** (aAS = 0.24) is comprised of 36 CLOGs mostly annotated as hypothetical or kinase proteins with 7 signaling related proteins, two heterocyst differentiation proteins, four membrane transporter related proteins, and two segregation proteins. Genes of module 3 can, with a few exceptions, only be found in filamentous cyanobacterial strains, indicating a role of these genes in filamentous growth. Likewise, **module 4** (aAS = 0.11) combines 30 CLOGs that are solely associated to filamentous cyanobacteria capable of differentiating to heterocysts. The majority of CLOGs in the module lack a specific annotation with only few exceptions, including cytochrome b6f subunit PetM or the heterocyst differentiation protein PatN.

Multiple other modules reveal interesting associations of CLOGs, such as CRISPR-related proteins in module 54, 93, and 97, possible chemotaxis genes in module 62, phosphonate lyase related proteins in module 71, and six transposases in module 50. To facilitate further analysis, we therefore provide the SimilarityViewer. The viewer includes the complete dataset of co-occurrence and allows the exploration of the co-occurrence neighborhood for any (cyanobacterial) gene of interest. See Fig D and E in S1 Text for details. Overall, we conclude that the analysis of co-occurrences using a network perspective reveals known functional associations, and thereby establishes a suitable tool to generate novel hypotheses about putative functional roles of genes.

## Discussion

We used comparative genome analysis to investigate the cyanobacterial pan-genome inferred from 77 strains whose complete genome sequence is available. Our focus was the co-occurrence of clusters of likely ortholog genes, denoted as CLOGs. The importance of co-occuring CLOGs and the use of phylogenetic profiles as a means to study functional relationships between genes is well recognized, in particular for prokaryotic genomes [23, 26, 53]. Earlier studies, however, only had access to a limited number of sequenced genomes [14]. Only recently, decreasing costs for nucleotide sequencing and dedicated initiatives to increase coverage of the cyanobacterial phylum [4] have resulted in an increased number of fully sequenced cyanobacterial genomes. The number of 77 fully sequences genomes considered here is already close to the recommended number of ∼ 100 genomes after which the inclusion of additional genomes only yields diminishing returns [54].

Whereas earlier studies typically focused on horizontal gene transfer [55], gene essentiality [15] or natural product synthesis [2], we were specifically interested in the co-occurrence of CLOGs as a tool to understand functional relationships between ortholog genes and environmental adaptations of cyanobacteria. The initial analysis of co-occurring CLOGs showed that (i) co-occurrences are indeed highly indicative of functional relationships, (ii) co-occurrence does not imply co-localization of the respective genes on the genome, and (iii) the analysis pair-wise co-occurrence is not sufficient to capture groups of CLOGs that are functionally related.

We introduced a network-based approach that allowed us to identify *modules* of co-occurring CLOGs. Our results showed that such modules indeed often suggest functional relationships. Straightforward examples include known operon-like structures and enzymes that catalyze sequential steps in metabolic pathways. Beyond these straightforward examples, modules can often be associated with specific cellular functions. Relevant examples are the assembly of gas vesicles proteins and the biosynthesis of molybdopterin, among several others. Detailed analysis revealed that individual modules exhibit high functional coherence and provide useful insight into the functional neighborhood of genes.

We hypothesize that the high functional coherence observed for the extracted modules is also a consequence of the restriction to the similar-yet-diverse cyanobacterial phylum: cyanobacteria form a distinct phylogenetic clade and show enormous diversity in their environments (with respect to temperature, salt concentration, humidity), cell shapes (single celled, filamentous), and metabolic capabilities (hydrogen production, diazotrophy). Yet cyanobacteria also share, with only few exceptions, a basic metabolic lifestyle: photoautotrophic growth using oxygenic photosynthesis as a primary source of energy and redox potential. We argue that this similar-yet-diverse nature of cyanobacterial growth represents an ideal test case to evaluate gene co-occurrences with respect to putative functional relationships.

In particular, earlier studies of the bacterial pan-genome either focused on a set of very closely related organisms, such as a set of 8 commensal and 21 pathogenic E. coli strains [56]. Or, vice versa, considered the bacterial pan-genome in its entirety [53]. We argue that the former approach typically lacks the necessary diversity to associate genetic content to particular cellular functions: the functional diversity of the considered set of very closely related organisms does not manifest itself in the presence or absence of individual genes. In latter approach, involving a vast number of unrelated bacterial species, possible functional relationships are easily obscured by the diversity of lifestyles and metabolic functions within the set of considered species. For example, a comparison with the results of a recent analysis reporting gene-gene co-occurrence across ∼600 bacterial species suggests that the modules identified here are far more specific and typically relate to aspects of cyanobacterial functioning and growth, such as nitrogen fixation or formation of gas vesicles—whereas the previous global analysis primarily revealed examples related to co-occurrence of enzymes related to few basic metabolic pathways [53].

Previous literature reported contradicting results whether the inclusion of additional (unrelated) genomes necessarily improves predictive power. A recent study found that a maximally diverse set of genomes always outperforms any more narrow set [54]. Therein predictive accuracy was computationally evaluated using predictions of GO terms. In contrast, other studies found that inclusion of parasitic, pathogenic and closely related genomes resulted in no improvement or even a drop in predictive performance [25], here measured by co-occurrence in the same KEGG pathways. While automated measures are a necessity for large-scale computational evaluation, the complexity of the modules identified here also indicates the limitations of such measures. We conjecture that different assessments of predictive power might also favor different sets of genomes. We argue that practical applications should involve a manual analysis—which requires easily accessible computational tools to explore the co-occurrence landscape of CLOGs. To this end, we provide the *SimilarityViewer* to explore and identify gene-gene co-occurrences beyond the examples discussed in the main text (Fig D and E in S1 Text). The toolbox is available for MATLAB (The MathWorks, Inc) as well as a stand-alone application for Mac, Linux, and Windows [http://sourceforge.net/p/similarityviewer/]. With the increasing number of fully sequences genomes, the analysis of co-occurrence will undoubtedly become a highly value approach to provide novel hypotheses for putative gene functions—beyond sequence similarity and co-localization.

## Materials and methods

### Acquisition of genomic data

We searched the NCBI Genome database (http://www.ncbi.nlm.nih.gov/genome/) for cyanobacterial entries and selected all fully sequenced and assembled strains. We further included all associated plasmid sequences, as well as the recently annotated *Escherichia coli* O111:H (denoted as *E. coli*). In total 78 chromosomes and 136 plasmids were sourced from the NCBI Genome database (January 17, 2015) as listed in S3 Table. An a brief description all strains and their environmental background is provided in S4 Table. A phylogenetic tree (Fig F in S1 Text) was constructed by extracting the 16S ribosomal RNA sequences of all genomes. Pair wise distances were calculated using the distance model by Jukes and Cantor [57] and the BLOSUM62 scoring matrix. The tree was constructed with the seqlinkage function by MATLAB using the default parameters. As expected, the only non-photosynthetic organism *E. coli* appears as an outgroup.

### Cluster of likely orthologous genes (CLOGs)

Identification of orthologous genes was done as previously described in Beck et al. [14]. Following an all-against-all BLASTp comparison, the bidirectional hit rate (BHR) between all gene pairs *a* (from genome A) and *b* (from genomes B) is defined as

$$BHR=[\frac{S_{a,b}}{S_{a}^{bestB}}]\times [\frac{S_{b,a}}{S_{b}^{bestA}}],$$ (1)

where *S*_*a*,*b*_ is the BLASTp score of *a* against *b* and $S_{a}^{bestB}$ is the best score of *a* against any gene in genome B (including *b*). The *BHR* = 1 for all mutually best hits and is lower otherwise. All gene pairs with *BHR* > 0.95 are grouped together. To avoid weakly connected groups, genes in each group were clustered according to their mutual BLASTp score using the UPGMA (unweighted pair group method with arithmetic mean) and a cut-off of 20. In the following, these clusters are referred to as CLOGs (Cluster of Likely Orthlogous Genes). Genes within each cluster are assumed to be orthologous. The method was previously evaluated and compared against other available toolboxes and databases to identify groups of ortholog genes and yields similar results [14]. We note that CLOGs may also only consist of a single gene (‘singletons’) if no ortholog (or paralog) is detected.

### Similarity of CLOGs and modules of co-occurrence

The pair-wise co-occurrence of CLOGs was evaluated in a two-step process. For all 1.7 × 10^9^ pairs of CLOGs, a right-sided Fisher’s exact test was calculated. P-values were corrected for multiple testing using the method by Benjamini and Yekutieli [33] with an excepted false discovery rate (FDR) of FDR = 0.01. The critical p-value was below 1.43*e*^−6^. For all significantly correlated pairs of CLOGs *i* and *j*, a similarity index (SI) was computed as

SI(i,j)=AMI(i,j)×(1-CI(i∩j,t)), (2)

where *AMI*(*i*, *j*) denotes the adjusted mutual information between the phylogenetic profiles of the CLOGs *i* and *j*. The AMI is a variant of the mutual information that is adjusted for lopsided frequencies, see Vinh et al. [58] for details. The AMI ranges between *AMI* = 0 for uncorrelated and *AMI* = 1 for fully correlated pairs. The Consistency Index *CI*(*i* ∩ *j*, *t*) measures the consistency of the 16S rRNA phylogenetic tree *t* (Fig F in S1 Text) to the set of strains participating in both CLOGs *i* and *j* [59]. The CI adjusts for the fact that co-occurrence is biased by phylogenetic proximity, hence the SI is “phylogeny-aware” [26]. That is, links between CLOGs that co-occur in phylogenetically closely related genomes (CI ≈ 1) are assigned less weight compared to links between CLOGS that co-occur in phylogenetically distant genomes (CI ≪ 1). E. coli and *Cyanobacterium* UCYN-A were not considered when calculating the CI. Anti-correlation between two CLOGs was quantified using the same method but using a left-sided Fisher’s exact test. Correction for multiple testing yielded a critical p-value of 4.42 × 10^−7^. The consistency index was not computed for anti-correlated CLOGs and therefore set to zero. The adjusted mutual information remains a positive value with plus one for fully anti-correlated pairs.

To extract modules of co-occuring CLOGs we consider CLOGs as nodes in a network, interconnected by weighted links. For any pair of CLOGs whose co-occurrence is statistically significant and whose SI exceeds a threshold *ξ*_*t*_, the weight *w*(*i*, *j*) of the link was assigned to a value proportional to the SI,

$$w\left(i,j\right)=\text{max}(0,SI\left(i,j\right)-\xi_{t}).$$ (3)

The heuristic parameter-free algorithm by Blondel et al. [35] was utilized to identify modules of co-occurring CLOGs. See Fig G and H in S1 Text for a detailed analysis of the modules properties.

### Computation of genomic adjacency

The adjacency score AS(*s*) represents the proximity of genes contained within a module for an individual strain *s*. To estimate the AS, the respective genes are ordered according to their position on the chromosome or plasmids. Two (neighboring) genes are defined to be in close proximity if less than 10 annotated open reading frames separate their loci. By definition, genes on different chromosomes/plasmids are not in close proximity. The AS for strain *s* is then defined as:

$$AS\left(s\right)=\frac{1}{n-1}\sum_{i=2}^{n}\{\begin{array}{cc} 1 & \text{if}\;\text{gene}{}_{i-1}\;\text{and}\;\text{gene}{}_{i}\;\text{in}\;\text{close}\;\text{proximity}\\ 0 & \text{otherwise} \end{array},$$ (4)

where *n* is the number of genes within the module. The sum runs over all *n*−1 (ordered) pairs of genes. The AS ranges between AS = 1 if all (neighboring) genes are separated by less than 10 annotated open reading frames and AS = 0 is no pair of (neighboring) genes is closer than ten annotated open reading frames. The average AS (aAS) of a module is then computed as the average AS(*s*) over all strains *s* that have least two genes within all CLOGs that comprising the module. The measure yields similar results than more traditional measures (see Fig I in S1 Text).

## Supporting information

S1 Text A pdf file with supporting text. The pdf contains a additional figures, a brief analysis of anti-correlated CLOGs, a more detailed analysis of co-occurrence versus genomic adjacency, as well as a tutorial of the SimilarityViewer.
(PDF)

S1 Table Table of correlated CLOGs sorted by p-value of Fisher’s exact test. Each line of this tab-separated text file corresponds to one pair of CLOGs and includes the CLOG numbers as well as the p-value obtained by Fisher’s exact test, the p-value corrected for multiple testing, the adjusted mutual information, and the consistency index based on the 16S phylogenetic tree. Rows are sorted by the uncorrected p-value. Pairs of CLOG with an uncorrected p-value lower than 0.01 are omitted.
(ZIP)

S2 Table Table of all CLOGs sorted according to modules. Table of all CLOGs sorted by their assignment to modules. For each CLOG, the table contains module number, number of the CLOG, all assigned genes sorted by strains, most common annotation, as well as all assigned reactions and EC numbers. Multiple entries in cells are delimited by a tilde (“~”).
(ZIP)

S3 Table List of chromosomes and plasmids. The PDF file enlists all 78 chromosome and 136 plasmids considered in this study. The GenBank accession ID links every sequence to the according entry in the GenBank database of the National Center for Biotechnology Information (NCBI) [http://www.ncbi.nlm.nih.gov/genbank/].
(PDF)

S4 Table Genomic and environmental information for all strains analyzed in this study. In this table, we provide genomic and growth information for each strain including natural habitat, morphology (sections I-V, according to [61]), number of chromosomes & plasmids, number of ORFs, genome size (in megabase pairs), G+C content (in percent), fraction of DNA in ORFs (in percent), number of CLOGs, number of core CLOGs, number of shared CLOGs, number of unique CLOGs, and number of CLOGs with assigned metabolic function. We also extracted from literature the strains’ ability to fixate atmospheric nitrogen. Literature data disagreeing with the findings in our study (strain has no orthologs in module 9, composed of CLOGs mostly associated to nitrogenase) is marked with an asterisk. The last column lists various information concerning habitat, metabolism, symbiosis, and particular features of the strains. Organisms of the genus *Prochlorococcus* are annotated with the water depth at which the according strain was found, and their adaptation to high light (HL) or low light (LL). If not noted otherwise, data regarding the structural section was extracted from [4], while information regarding habitat, nitrogen fixation, and general properties was extracted from [62].
(PDF)
