# Supplementary material for: Modules of co-occurrence in the cyanobacterial pan-genome reveal functional associations between groups of ortholog genes
Source: PLoS Genet. 2018 Mar 9;14(3):e1007239. doi: 10.1371/journal.pgen.1007239 (PMC5862535; doi:10.1371/journal.pgen.1007239)
Supplement: S1 Text — The pdf contains a additional figures, a brief analysis of anti-correlated CLOGs, a more detailed analysis of co-occurrence versus genomic adjacency, as well as a tutorial of the SimilarityViewer. (PDF) [file pgen.1007239.s001.pdf]

Supporting Text 1: SUPPLEMENTAL TEXT AND FIGURES

Modules of co-occurrence in the cyanobacterial pan-genome reveal functional associations between groups of ortholog genes

Christian Beck, Henning Knoop, and Ralf Steuer

Humboldt-Universität zu Berlin, Institut für Biologie, FachInstitut für Theoretische Biologie (ITB), Invalidenstr. 43, 10115 Berlin, Deutschland

Correlation and anticorrelation of CLOGs

We focused on the analysis of functional relationships of correlating genes. These are genes occurring in a similar subset of organisms. In addition to co-occurrence discussed in the main text, we also analyzed anti-correlation of genes. These pairs of CLOGs are associated to mutually excluding subsets of organisms. In contrast to co-occurrence, negative correlation is less common in our data. Using the left-tailed Fisher's exact test with the same correction method (Benjamini and Yekutieli 2001) and an identical false discovery rate of 0.01, we identified only 178408 anti-correlated pairs, or about 30% of the number of correlated pairs. Examples with low p-values include CLOGs 6775 (dethiobiotin synthase) and 8906 (hypothetical gene), CLOGs 4535 (alpha/beta hydrolase fold) and 6308 (helicase), as well as CLOGs 5269 (transporter membrane component) and 5503 (carboxysome associated protein). We could not detect systematic functional relations of negatively correlated CLOGs. By their nature, anti-correlated CLOGs cannot be grouped into modules of multiple CLOGs.

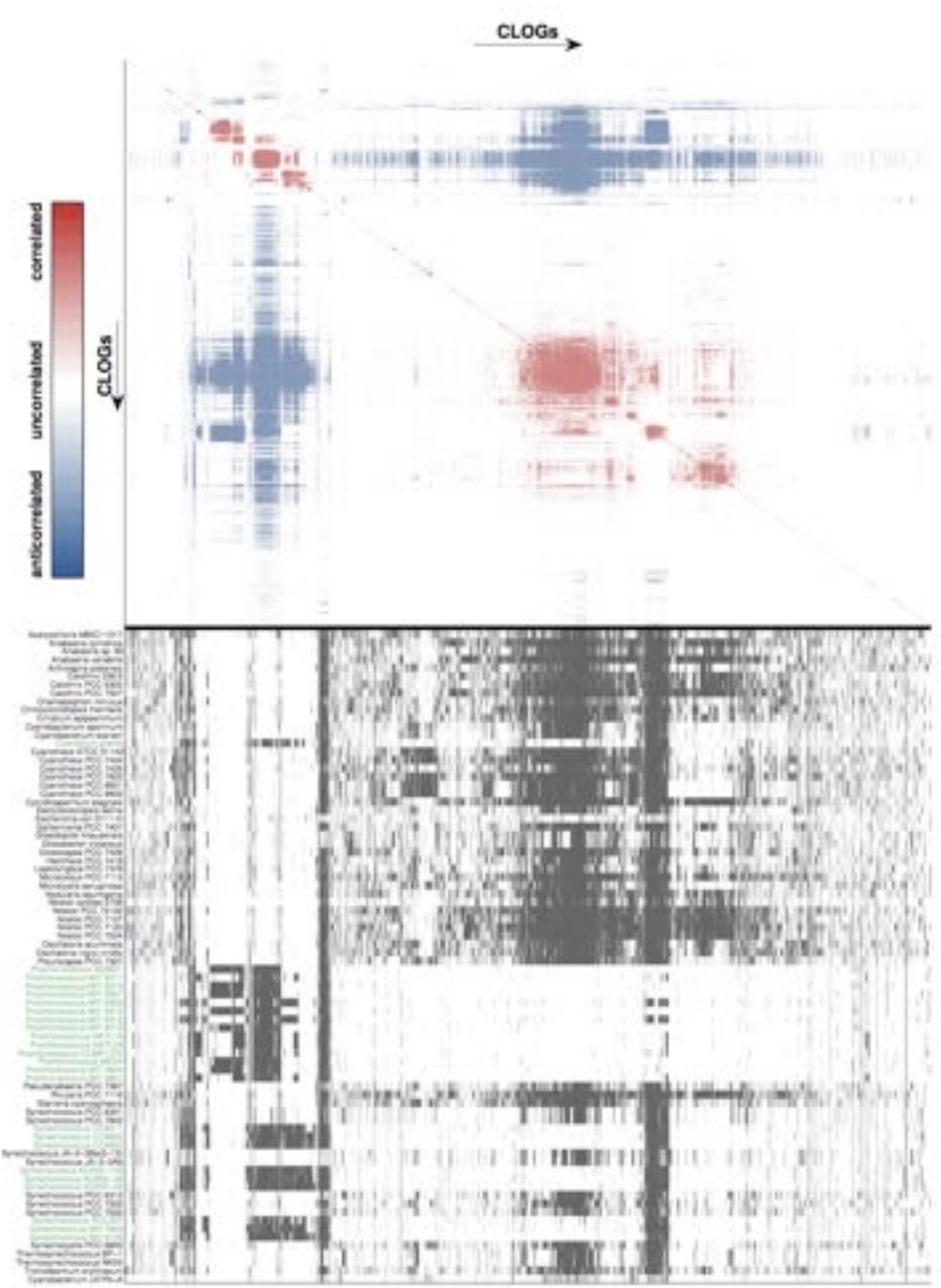

Figure A: Meta-structure of co-occurrence. Color-coded representation of the adjusted mutual information (AMI) between shared CLOGs indicating correlation and anti-correlation. CLOGs are

ordered using the UPGMA method with pairwise AMI as distance. Shades of red indicate positive correlation whereas blue indicates anti-correlation. The lower part shows the association of organisms to each CLOG, with alpha-cyanobacteria labeled in green. The structure of co-occurrence indicates a dominant bisection into CLOGs solely associated with alpha-cyanobacteria (upper/left side) or Beta-cyanobacteria (middle). Within these groups CLOGs are highly correlated. Strongest anti-correlation is found between pairs of CLOGs across both groups. CLOGs with no significant correlation to any other CLOG are omitted from the figure.

However, the data indicates a dominant association of most anti-correlated pairs of CLOGs to particular phylogenetic clades (Figure F), namely an exclusive association to either alpha-cyanobacteria or beta-cyanobacteria (Figure A). These two families are distinguished by the molecular structure of the RuBisCO proteins and carboxysomes, and have separated around one billion years ago. Alpha-cyanobacteria most likely acquired alpha-carboxysomes through horizontal gene transfer from proteobacteria (Badger and Price 2003; Whitehead et al. 2014). Above all other factors, this ancestral separation of alpha- and beta-cyanobacteria reflects the predominant differences of cyanobacterial genomes.

Strain-specific co-localization

In addition to investigating the module specific aAS, we analyzed the co-localization of correlated genes within each organism. However, when calculating the average AS for each strain, we could not identify any correlation with genome size or number of plasmids (Figure B). The rate of co-localization for small organisms with streamlined genomes (e.g. *Prochlorococcus*) was on par with large organisms incorporating multiple plasmids (e.g. *Acaryochloris marina*). Only when comparing the distribution of the AS of all genomes with the strain specific distribution we were able to identify small but significant differences between the genomes (Figure C). Using the Kolmogorow-Smirnov test we were able to identify 10 strains with a significantly lower rate of co-localization (p-value < 0.01). Among the cyanobacteria with the lowest p-value are C*yanobacterium aponinum* PCC 10605, *Cyanobacterium stanieri* PCC 7202, *Leptolyngbya* sp. PCC 7376 both strains of

Thermosynechococcus (BP-1 and NK55), as well as the model organism *Synechocystis* sp. PCC 6803. In these organisms the genome is more fragmented than in other cyanobacteria, making the identification of functionally related genes solely based on their genomic adjacency harder.

In contrast, we could not identify a single strain with a significantly more ordered genome. For some strains including E. coli, a model organism for the analysis of operons (Salgado et al. 2000; Gama-Castro et al. 2016) the average adjacency score is higher when compared to the background. However, the differences were not significant according to our test with a significance level of 0.01.

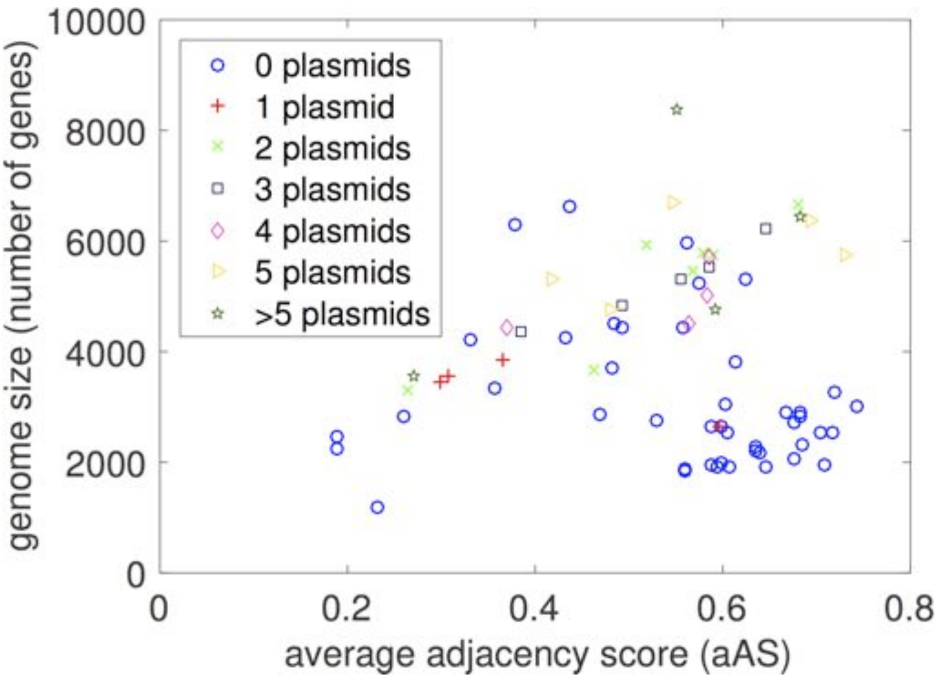

Figure B: Average adjacency score and genome size of all strains. The mean adjacency score was calculated by averaging the AS of all genes from a single organism co-occurring in any module. Neither the genome size nor the number of plasmids correlates with the average adjacency score, although plasmids are reported to possibly help in organizing functionally related genes (Jones et al. 1991).

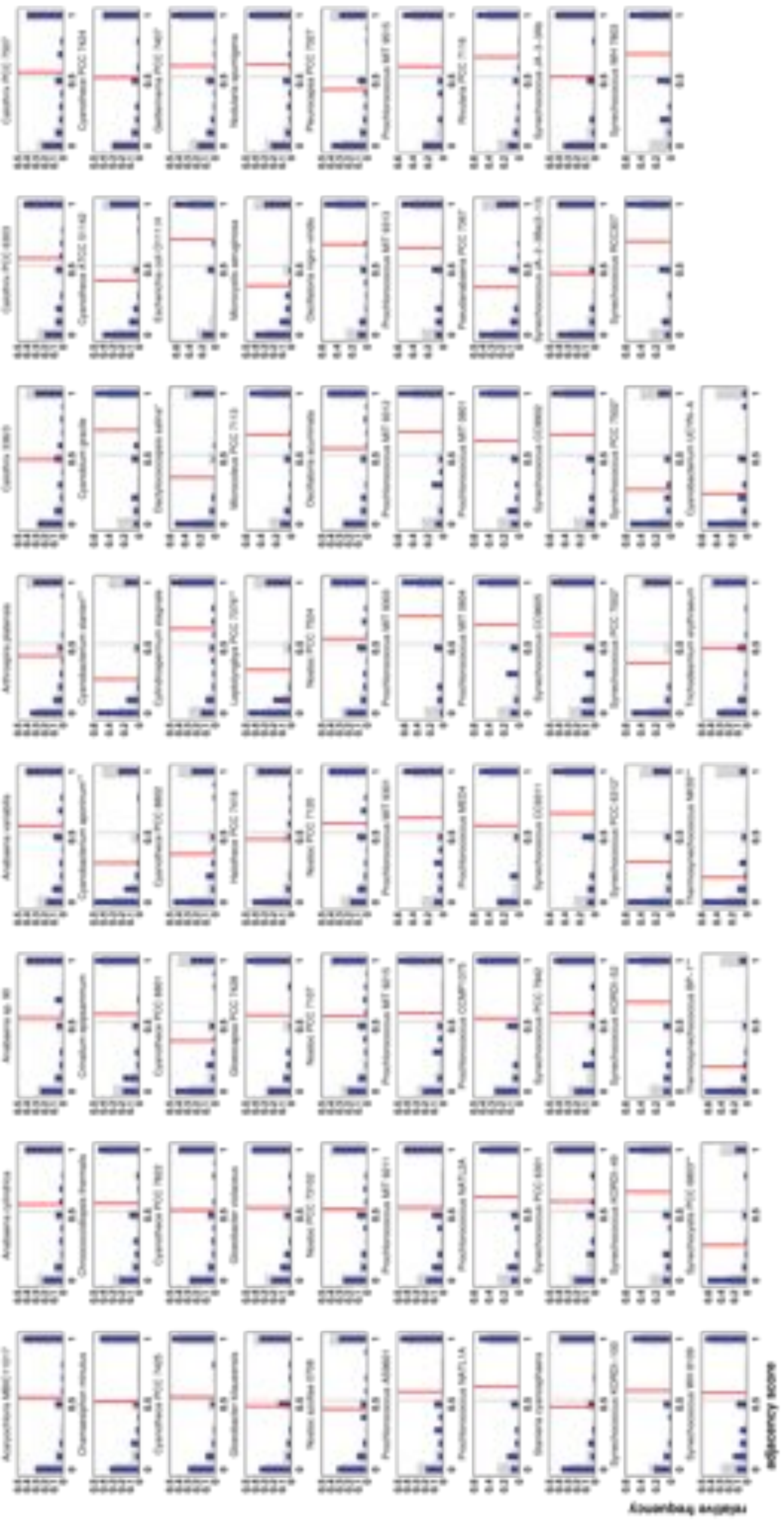

Figure C: Distribution of the genomic proximity for each strain. Each plot shows the distribution of the adjacency score (blue bars) for a specific bacterial strain as well as the background distribution across all strains (gray bars). Red and gray lines mark the mean adjacency score for the strain-specific and background distribution, respectively. Strains marked by asterisks show a distribution significantly different to the background distribution with p<0.01 (*) and p<0.001 (**) using the two-sided Kolmogorow-Smirnov-Test implemented in MATLAB. While the distribution of the adjacency score is similar to the background in most organisms, some strains show a significantly lower score – most notably *Cyanobacterium aponinum*, *Cyanobacterium stanieri*, both *Thermosynechococcus*, and the model organism *Synechocystis* PCC 6803. Organization of genes in these organisms seems to be more fragmented compared to other cyanobacteria. Adjacency in *Escherichia coli* on the other hand seems to be above average but is not significantly different to the cyanobacterial strains.

The CyanoCLOG Similarity Viewer

With the CyanoCLOG Similarity Viewer (CSV) we provide an easy to use software tool to browse and compare the CLOGs identified in this study. The tool is written in MATLAB (The MathWorks) and can be run directly from the MATLAB command line. Alternatively, a standalone version can run on computers without MATLAB, using MATLAB Runtime (MCR).

The CSV can be downloaded under [http://sourceforge.net/p/similarityviewer/]. Installation of the MATLAB command line version requires unpacking the zip file into a folder and running the “runSV.m” file from the MATLAB command line. This script will load the content of data.mat into the workspace and start the CSV. Alternatively, standalone versions are provided for Linux, Mac, and Windows to run on computers without MATLAB. These versions need the installation of the MCR, which will be done automatically when running the SimilarityViewer_install file appropriate for the operating system.

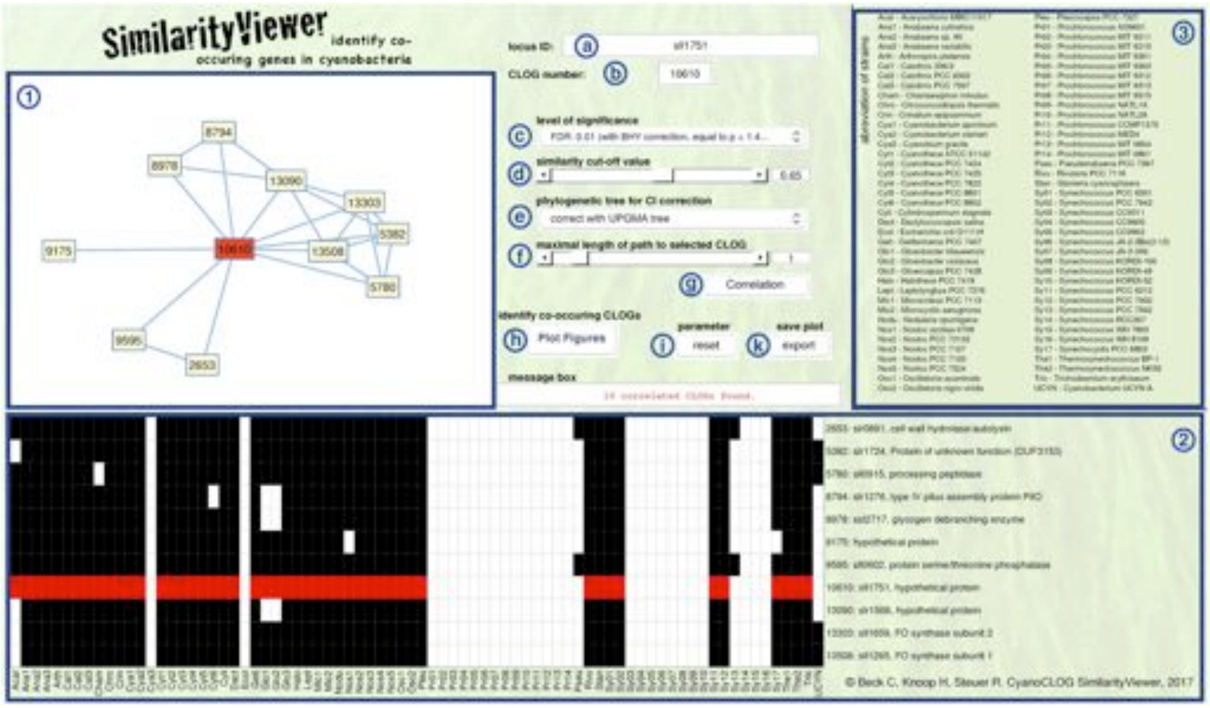

Figure D: Screenshot of the SimilarityViewer. After startup, the SimilarityViewer is opened in a single window, which is separated into 4 areas. The center area features all control elements for an easy search and comparison of CLOGs. There are two ways to specify a single CLOG. If a specific gene identifier is put into box (a) the program automatically searches for the corresponding CLOG and shows the CLOG number in box (b). Alternatively, the CLOG number can be given in box (b) directly. Drop-down menus and sliders (c) to (f) enable the adjustment of parameters for the similarity search. Button (g) allows to toggle between the search for correlated or anti-correlated CLOGs. The large main button (h) finally activates the search for (anti-)correlated CLOGs. The last two buttons reset all parameter to default values (i) and export the lower graph to a separate figure file (k). The message box at the lower end of the control panel shows error messages and varying information, depending on the last operation. The results of the search for correlated CLOs is presented in two plots. Plot number one on the upper left shows the network of all correlated CLOGs, where every edge connects two CLOGs identified as correlated on the basis of the current parameter set. The lower plot number two shows all correlated CLOGs and their assigned set of strains. Each line represents one CLOG, with a non-white box if the CLOG contains at least one gene from the organism listed on the x-axis. Each CLOG is labeled with the CLOG number, the most probable annotation, and - if applicable - the locus ID of the genes from the same organism as the gene given in (a). Clicking on one of the boxes shows the gene ID of the affected strain in the message box. In both plot one and two, the selected

CLOG shown in (b) is highlighted in red. Panel number 3 in the upper right gives the full name for the abbreviations in plot 2.

The SV is a simple to use graphical tool as depicted in Supplement figure D. To specify a CLOG one can simply type in the locus identifier of any gene into box (a), and the system will automatically identify the CLOG corresponding to this gene. Alternatively, the CLOG number can directly be entered into box (b). Four numeric parameters can be specified via sliders and drop-down menus:

(c): Select the significance level of the Fisher's exact test between two CLOGs. Two CLOGs with a larger test value will not be identified as correlated. Options are False Discovery Rate of 0.01 using the correction method by Benjamini and Yekutieli (Benjamini and Yekutieli 2001) (critical uncorrected p-Value ≈1.43E-6) and the uncorrected p-values of the Fisher's exact test 1E-5, 0.001, and 0.01. This parameter only has an effect for low AMI cut-off values.

(d): Adjust the cut-off value for the similarity. The similarity of two CLOGs is computed as S = AMI * (1-CI), where AMI is the adjusted mutual information and CI is the consistency index, measuring the consistency of co-occurence with the phylogenetic tree shown in Supplemental figure F. S can range from zero (no similarity) to 1 (highest correlation/anti-correlation). The cut-off can be set to any value between 0.2 and 1 using the slider or by entering a value. Pairs of CLOGs with similarity higher than this cutoff will be considered correlated.

(e): Select/deselect phylogenetic tree for consistency correction. Deselecting a tree will set the consistency index CI for the calculation of the similarity to zero. Thus, the resulting pairs of CLOGs will not be corrected for their phylogenetic relation.

(f): Maximal distance in network of correlated CLOGs. This parameter restricts the maximal distances of any CLOG to the selected CLOG within the computed network (upper left graph). For example: if the parameter is set to 1 the set of considered CLOGs is limited to CLOGs directly correlated to the base CLOG given in (b). If the parameter is set to 2 the set

of CLOGs also include CLOGs indirectly correlated to the base CLOG via one other CLOG. This parameter can be set to value between zero and ten. Large values can increase the calculation time considerably.

Below the numeric parameter controls, the SV features four buttons. Switch (g) toggles between correlation and anti-correlation of CLOGs. When searching for anti-correlated CLOGs, the network distance (f) is fixed at one and the consistency correction is deactivated. Main button (h) initiates the search for correlated CLOGs given the selected parameters and draws the correlation network as well as the CLOG plot in the bottom area. Button (i) resets all parameter to the default values, used to identify modules of co-occurrence in this study. The lower right button (k) saves the bottom graph (2) of CLOGs in an external file. An example is shown in Supplemental figure E. The SV utilizes the “export_fig” function provided by Yair Altman [https://www.mathworks.com/matlabcentral/fileexchange/23629-export-fig] to achieve a clean graphic export.

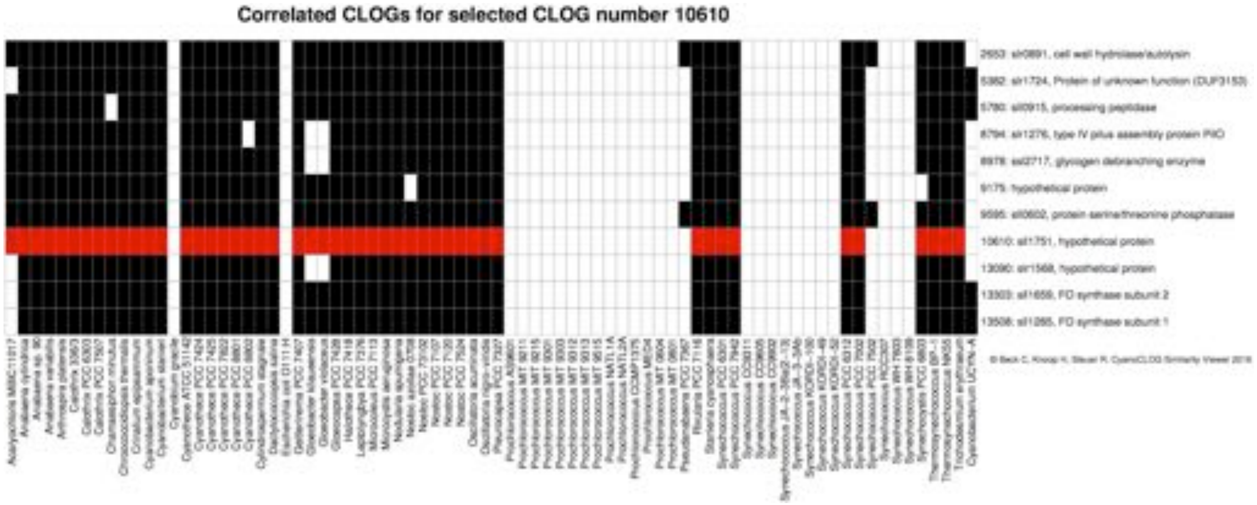

Figure E: Correlated CLOGs and associated organisms. Figure of correlated CLOGs and their associated bacterial strains as exported from the SimilarityViewer (SV). Each line represents one CLOG, with non-white boxes for every strain indicated on the x-axis that participates with at least one gene. Red boxes indicate the CLOG selected as base for the search of correlated CLOGs. On the right, all CLOGs are labeled with their number and most common annotation.

Calculation of the phylogenetic tree

The phylogenetic tree (Figure F) was constructed for all 78 strains by extracting the 16S ribosomal RNA sequences. Pair wise distances were calculated using the "seqpdist" function by MATLAB with the BLOSUM62 scoring matrix and the Maximum likelihood estimate for substitutions by Jukes and Cantor (Jukes and Cantor 1969). The tree was constructed with the "seqlinkage" function by MATLAB using the standard parameter. The only non-photosynthetic organism Escherichia coli appeared as outgroup. The group of α-cyanobacteria indicated by a magenta bar emerged as a distinct clade, confirming the general suitability of this method. This tree was subsequently used to compute the consistency index of co-occurrences between CLOGs.

Figure F also indicates the genome size for each organism represented by the number of associated CLOGs. The genomes are divided into core CLOGs (associated to "all" strains but optional in E. coli and cyanobacterium UCYN-A), shared CLOGs (more than one organism associated), and unique CLOGs (only one associated organism).

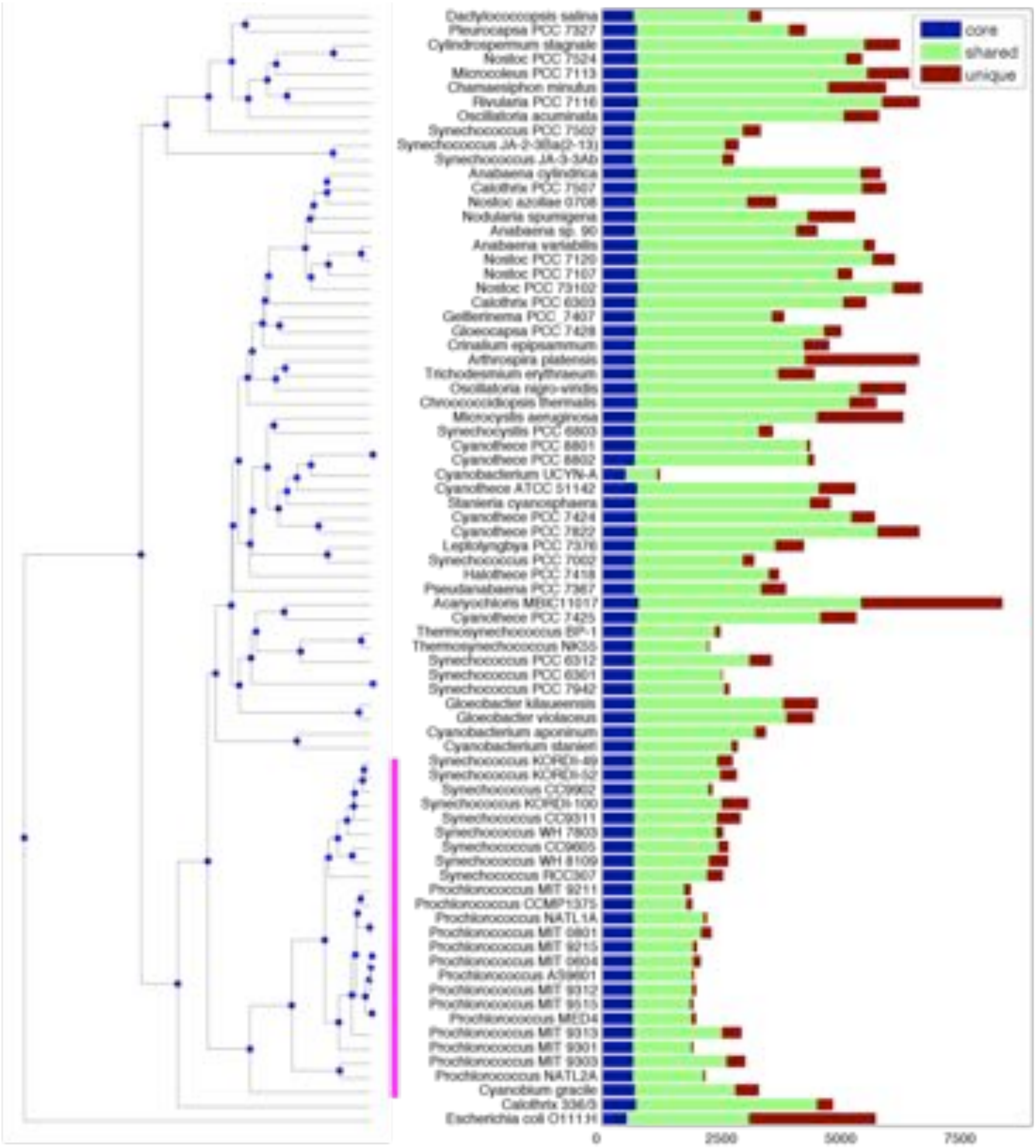

Figure F: Phylogeny and genomic properties of all 78 bacterial stains. The phylogenetic tree on the left shows the lineage of all 78 bacteria based on their 16S ribosomal RNA. Pair-wise distances were calculated with the method by Jukes and Cantor (Jukes and Cantor 1969) using the BLOSUM62 scoring matrix. The tree was constructed with the “seqlinkage” function of MATLAB with standard parameter. *Escherichia coli*, the only non-photosynthetic organism naturally appeared as outgroup. The vertical magenta bar in the middle marks the cluster of alpha-cyanobacteria. The right side shows size and composition of the respective strains. The size of the bars indicates the total number of genes in the genomes divided into core genes (blue), shared genes (green), and unique genes (red). The core genes are defined as genes found in all strains but optional in the reduced Cyanobacterium UCYN-A and Escherichia Coli. The absolute number of core genes per strain can vary due to gene duplications in some genomes.

Modules of co-occurring genes

Utilizing a network based approach, we grouped CLOGs into modules of co-occurring and therefore functionally related genes as explained in the main text. To investigate possible biases of the clustering method, a scatter plot was created to identify correlations between the sizes of modules with respect to the number of participating CLOGs as well as the number of participating strains (Figure G). Modules with only 2 participating CLOGs are indeed associated mostly with only few strains (the red diamonds indicate the median of the distributions). However, 2-member modules are not restricted to few participating strains but cover the full range of participating strains.

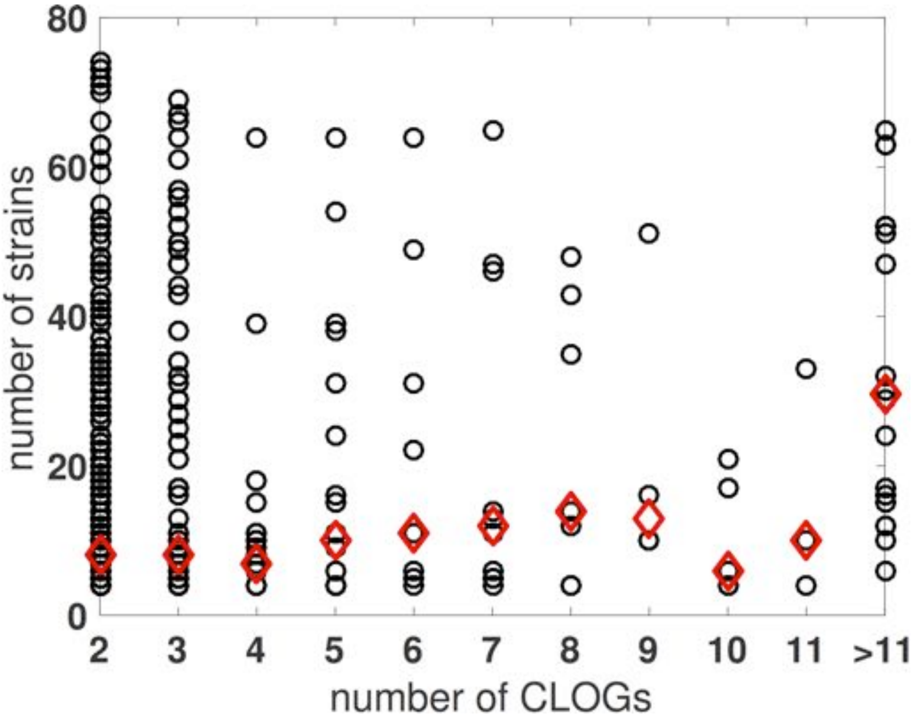

Figure G. Relationship between the number of participating strains versus the number of CLOGs associated with the identified modules of co-occurrence. Each circle of the scatter plot corresponds to a module and indicates the number of CLOGs a well as the number of associated strains. Red diamonds show the median of the data to indicate the weight of the distribution.

To investigate a possible bias, we further consider a histogram of the (relative) number of CLOGs associated with a given number of strains for the CLOGs grouped into modules versus the background of all CLOGs (the figure is truncated to include only CLOGs associated with > 3 strains, since no CLOG grouped into a module is associated with less than 4 strains). Fig. S6 shows that the relative frequency largely coincides. We note that due to weighting the similarity index (used to identify modules) with the phylogenetic diversity of the associated strains (calculated as the consistency index CI), CLOGs associated to less than 4 four or more than 74 strains are not grouped into modules.

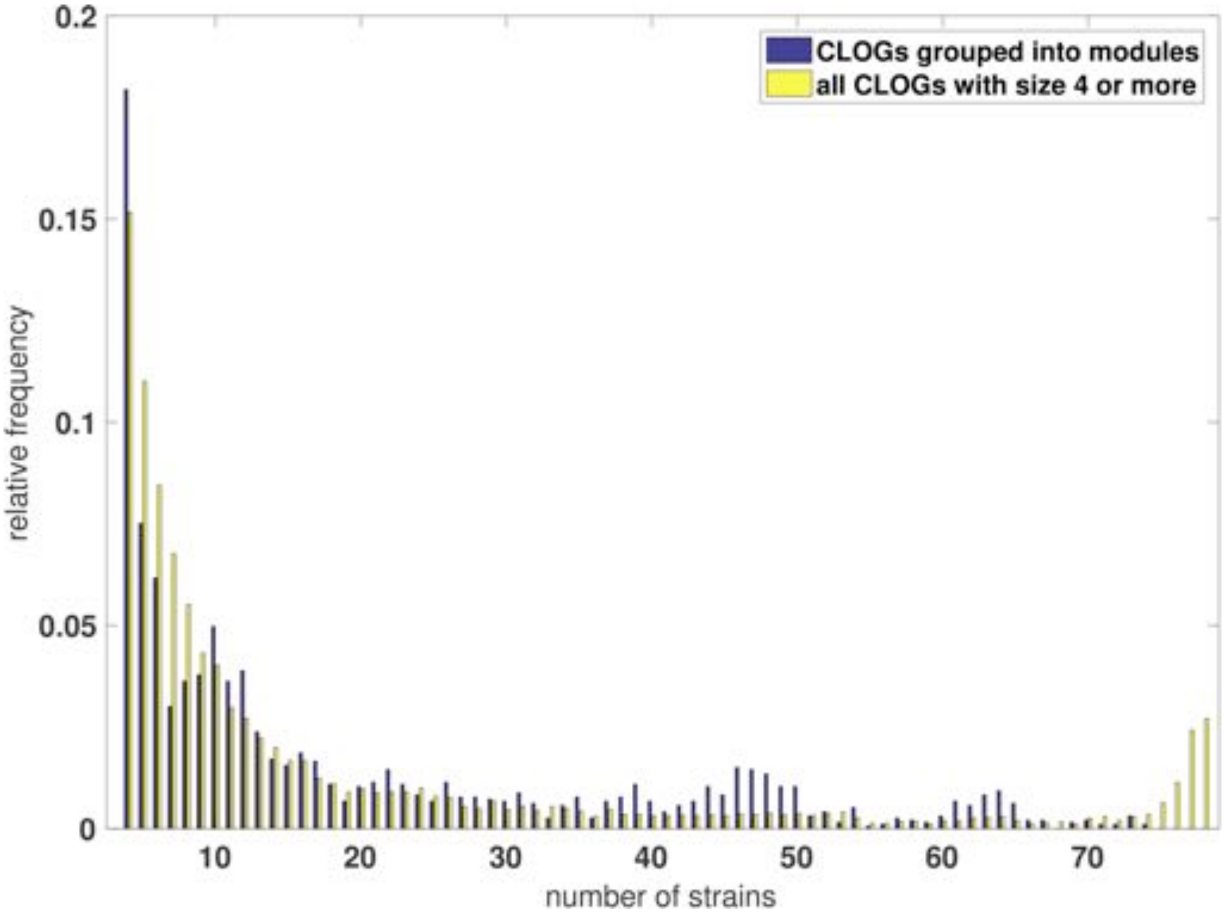

Figure H: Relative frequency of the number of assigned strains to all CLOGs and CLOGs grouped into modules. Each bar indicates the relative frequency of CLOGs grouped into modules associated to a specific number of strains (blue bars) or the relative frequency of all CLOGs (yellow bars). The plots only shows CLOGs with 4 or more associated strain since no module is associated with less than 4, or more than 74 strains. Both distributions are in good agreement and show no clear disparity for either small or large CLOGs with respect to the number of associated strains.

Co-localization of co-occurring genes

We compared the averaged adjacency score (aAS) used in this work with a previously published gene neighbor method that was published by Bowers and colleagues (Bowers et al. 2004) and scored among the best in a systematic comparison of genome context methods (Ferrer et al. 2010). This method, denoted as *gn-norm-lnX* by Ferrer at al., rates the gene neighborhood of two genes and their homologues in a given set of organisms. For every pair of the gene homologues within a genome, the probability that fewer ORFs are located between the two genes than the observed d_I is computed as

$$p_i = \frac{2d_i}{N_i - 1}, \quad (1)$$

where N_i denotes the total number of genes on that chromosome. The gene neighbor score S over all the homologues of the two genes (in our work referred to as CLOGs) is then computed as the negative sum of the logarithms of the probabilities, normalized by the number of organisms:

$$S = \frac{-\sum_{i=1}^{M} \log(p_i)}{M}, \quad (2)$$

with M being the number of genomes with homologues of both genes. To calculate the normalized gene neighbor score of one module, we averaged S for all possible pairs of CLOGs. As indicated in Figure I, the aAS shows a good correlation to the gene neighbor score. We opted in favor of calculating the aAS because it permits calculation of distances not only for pairs but groups of multiple CLOGs. Furthermore, this method allows for the comparison of gene co-localization between specific organisms.

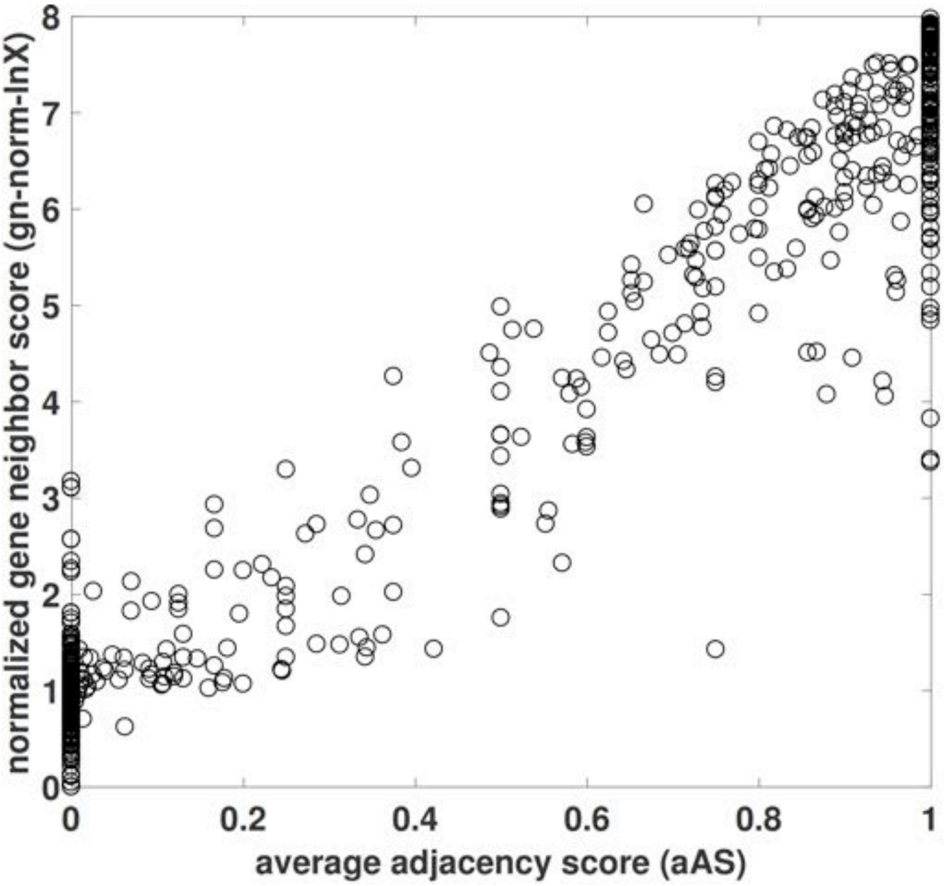

Figure I: Comparing the average adjacency score to the normalized gene neighbor score. Calculating for every module the average adjacency score (aAS) as well as the normalized neighbor score *gn-norm-lnX* (Ferrer et al. 2010) averaged over all pairs of CLOGs within that module reveals a good correlation of both measurements. Outliers such as module 371 with an aAS=0.75 but a gene neighbor score of only 1.42 can be attributed to pairs of genes on smaller plasmids, as the probability of two homologues being in close neighborhood is dependent on the chromosome size N_i (formula (1)).

REFERENCES

Badger MR, Price GD. 2003. CO2 concentrating mechanisms in cyanobacteria: molecular components, their diversity and evolution. *Journal of experimental botany* **54**: 609-622.

Benjamini Y, Yekutieli D. 2001. The control of the false discovery rate in multiple testing under dependency. *Ann Stat* **29**: 1165-1188.

Bowers PM, Pellegrini M, Thompson MJ, Fierro J, Yeates TO, Eisenberg D. 2004. Prolinks: a database of protein functional linkages derived from coevolution. *Genome biology* **5**: R35.

Ferrer L, Dale JM, Karp PD. 2010. A systematic study of genome context methods: calibration, normalization and combination. *BMC bioinformatics* **11**: 493.

Gama-Castro S, Salgado H, Santos-Zavaleta A, Ledezma-Tejeida D, Muniz-Rascado L, Garcia-Sotelo JS, Alquicira-Hernandez K, Martinez-Flores I, Pannier L, Castro-

Mondragon JA et al. 2016. RegulonDB version 9.0: high-level integration of gene regulation, coexpression, motif clustering and beyond. *Nucleic acids research* **44**: D133-143.

Jones JG, Young DC, DasSarma S. 1991. Structure and organization of the gas vesicle gene cluster on the Halobacterium halobium plasmid pNRC100. *Gene* **102**: 117-122.

Jukes TH, Cantor CR. 1969. Evolution of protein molecules. *Mammalian protein metabolism* **3**: 132.

Salgado H, Moreno-Hagelsieb G, Smith TF, Collado-Vides J. 2000. Operons in Escherichia coli: genomic analyses and predictions. *Proceedings of the National Academy of Sciences of the United States of America* **97**: 6652-6657.

Whitehead L, Long BM, Price GD, Badger MR. 2014. Comparing the in vivo function of alpha-carboxysomes and beta-carboxysomes in two model cyanobacteria. *Plant physiology* **165**: 398-411.
