# Supplementary material for: Modules of co-occurrence in the cyanobacterial pan-genome reveal functional associations between groups of ortholog genes
Source: PLoS Genet. 2018 Mar 9;14(3):e1007239. doi: 10.1371/journal.pgen.1007239 (PMC5862535; doi:10.1371/journal.pgen.1007239)
Supplement: S3 Table — The PDF file enlists all 78 chromosome and 136 plasmids considered in this study. The GenBank accession ID links every sequence to the according entry in the GenBank database of the National Center for Biotechnology Information (NCBI) [http://www.ncbi.nlm.nih.gov/genbank/]. (PDF) [file pgen.1007239.s004.pdf]

Name of strain [Abreviation]	GenBank accession ID	Definition
Acaryochloris marina MBIC11017 [Acar]	CP000828	Acaryochloris marina MBIC11017, complete genome.
	CP000838	Acaryochloris marina MBIC11017 plasmid pREB1, complete sequence.
	CP000839	Acaryochloris marina MBIC11017 plasmid pREB2, complete sequence.
	CP000840	Acaryochloris marina MBIC11017 plasmid pREB3, complete sequence.
	CP000841	Acaryochloris marina MBIC11017 plasmid pREB4, complete sequence.
	CP000842	Acaryochloris marina MBIC11017 plasmid pREB5, complete sequence.
	CP000843	Acaryochloris marina MBIC11017 plasmid pREB6, complete sequence.
	CP000844	Acaryochloris marina MBIC11017 plasmid pREB7, complete sequence.
	CP000845	Acaryochloris marina MBIC11017 plasmid pREB8, complete sequence.
	CP000846	Acaryochloris marina MBIC11017 plasmid pREB9, complete sequence.
Anabaena cylindrica PCC 7122 [Ana1]	CP003659	Anabaena cylindrica PCC 7122, complete genome.
	CP003660	Anabaena cylindrica PCC 7122 plasmid pANACY.01, complete sequence.
	CP003661	Anabaena cylindrica PCC 7122 plasmid pANACY.02, complete sequence.
	CP003662	Anabaena cylindrica PCC 7122 plasmid pANACY.03, complete sequence.
	CP003663	Anabaena cylindrica PCC 7122 plasmid pANACY.04, complete sequence.
	CP003664	Anabaena cylindrica PCC 7122 plasmid pANACY.05, complete sequence.
	CP003665	Anabaena cylindrica PCC 7122 plasmid pANACY.06, complete sequence.
Anabaena sp. 90 [Ana2]	CP003284	Anabaena sp. 90 chromosome chANA01, complete sequence.
	CP003285	Anabaena sp. 90 chromosome chANA02, complete sequence.
	CP003286	Anabaena sp. 90 plasmid pANA01, complete sequence.
	CP003287	Anabaena sp. 90 plasmid pANA02, complete sequence.
	CP003288	Anabaena sp. 90 plasmid pANA03, complete sequence.
Anabaena variabilis ATCC 29413 (Anabaena flos-aquae UTEX 1444) [Ana3]	CP000117	Anabaena variabilis ATCC 29413, complete genome.
	CP000118	Anabaena variabilis ATCC 29413 incision element, complete sequence.
	CP000119	Anabaena variabilis ATCC 29413 plasmid A, complete sequence.
	CP000120	Anabaena variabilis ATCC 29413 plasmid B, complete sequence.
	CP000121	Anabaena variabilis ATCC 29413 plasmid C, complete sequence.
Arthrospira platensis NIES-39 [Arth]	AP011615	Arthrospira platensis NIES-39, 19 ordered pieces.
Calothrix sp. 336/3 [Cal1]	CP011382	Calothrix sp. 336/3, complete genome.
	CP011383	Calothrix sp. 336/3 plasmid unnamed1, complete sequence.
	CP011384	Calothrix sp. 336/3 plasmid unnamed2, complete sequence.
	CP011385	Calothrix sp. 336/3 plasmid unnamed3, complete sequence.
Calothrix sp. PCC 6303 [Cal2]	CP003610	Calothrix sp. PCC 6303, complete genome.
	CP003611	Calothrix sp. PCC 6303 plasmid pCAL6303.01, complete sequence.
	CP003612	Calothrix sp. PCC 6303 plasmid pCAL6303.02, complete sequence.
	CP003613	Calothrix sp. PCC 6303 plasmid pCAL6303.03, complete sequence.
Calothrix sp. PCC 7507 [Cal3]	CP003943	Calothrix sp. PCC 7507, complete genome.
Chamaesiphon minutus PCC 6605 [Cham]	CP003600	Chamaesiphon minutus PCC 6605, complete genome.
	CP003601	Chamaesiphon minutus PCC 6605 plasmid pCHA6605.01, partial sequence.
	CP003602	Chamaesiphon minutus PCC 6605 plasmid pCHA6605.02, complete sequence.
Chroococcidiopsis thermalis PCC 7203 [Chro]	CP003597	Chroococcidiopsis thermalis PCC 7203, complete genome.
	CP003598	Chroococcidiopsis thermalis PCC 7203 plasmid pCHRO.01, complete sequence.
	CP003599	Chroococcidiopsis thermalis PCC 7203 plasmid pCHRO.02, complete sequence.
Crinalium epipsammum PCC 9333 [Crin]	CP003620	Crinalium epipsammum PCC 9333, complete genome.
	CP003621	Crinalium epipsammum PCC 9333 plasmid pCRI9333.01, complete sequence.
	CP003622	Crinalium epipsammum PCC 9333 plasmid pCRI9333.02, complete sequence.
	CP003623	Crinalium epipsammum PCC 9333 plasmid pCRI9333.03, complete sequence.
	CP003624	Crinalium epipsammum PCC 9333 plasmid pCRI9333.04, complete sequence.
	CP003625	Crinalium epipsammum PCC 9333 plasmid pCRI9333.05, complete sequence.
	CP003626	Crinalium epipsammum PCC 9333 plasmid pCRI9333.06, complete sequence.
	CP003627	Crinalium epipsammum PCC 9333 plasmid pCRI9333.07, complete sequence.
	CP003628	Crinalium epipsammum PCC 9333 plasmid pCRI9333.08, complete sequence.
Cyanobacterium aponinum PCC 10605 [Cya1]	CP003947	Cyanobacterium aponinum PCC 10605, complete genome.
	CP003948	Cyanobacterium aponinum PCC 10605 plasmid pCYAN10605.01, complete seq.
Cyanobacterium stanieri PCC 7202 [Cya2]	CP003940	Cyanobacterium stanieri PCC 7202, complete genome.
Cyanobium gracile PCC 6307 [Cya3]	CP003495	Cyanobium gracile PCC 6307, complete genome.

Cyanothece sp. ATCC 51142 [Cyt1]	CP000806	Cyanothece sp. ATCC 51142 circular chromosome, complete sequence.
	CP000807	Cyanothece sp. ATCC 51142 linear chromosome, complete sequence.
	CP000808	Cyanothece sp. ATCC 51142 plasmid A, complete sequence.
	CP000809	Cyanothece sp. ATCC 51142 plasmid B, complete sequence.
	CP000810	Cyanothece sp. ATCC 51142 plasmid C, complete sequence.
	CP000811	Cyanothece sp. ATCC 51142 plasmid D, complete sequence.
Cyanothece sp. PCC 7424 [Cyt2]	CP001291	Cyanothece sp. PCC 7424, complete genome.
	CP001292	Cyanothece sp. PCC 7424 plasmid pP742401, complete sequence.
	CP001293	Cyanothece sp. PCC 7424 plasmid pP742402, complete sequence.
	CP001294	Cyanothece sp. PCC 7424 plasmid pP742403, complete sequence.
	CP001295	Cyanothece sp. PCC 7424 plasmid pP742404, complete sequence.
	CP001296	Cyanothece sp. PCC 7424 plasmid pP742405, complete sequence.
	CP001297	Cyanothece sp. PCC 7424 plasmid pP742406, complete sequence.
Cyanothece sp. PCC 7425 [Cyt3]	CP001344	Cyanothece sp. PCC 7425, complete genome.
	CP001345	Cyanothece sp. PCC 7425 plasmid pP742501, complete sequence.
	CP001346	Cyanothece sp. PCC 7425 plasmid pP742502, complete sequence.
	CP001347	Cyanothece sp. PCC 7425 plasmid pP742503, complete sequence.
Cyanothece sp. PCC 7822 [Cyt4]	CP002198	Cyanothece sp. PCC 7822, complete genome.
	CP002199	Cyanothece sp. PCC 7822 plasmid Cy782201, complete sequence.
	CP002200	Cyanothece sp. PCC 7822 plasmid Cy782202, complete sequence.
	CP002201	Cyanothece sp. PCC 7822 plasmid Cy782203, complete sequence.
	CP002202	Cyanothece sp. PCC 7822 plasmid Cy782204, complete sequence.
	CP002203	Cyanothece sp. PCC 7822 plasmid Cy782205, complete sequence.
	CP002204	Cyanothece sp. PCC 7822 plasmid Cy782206, complete sequence.
Cyanothece sp. PCC 8801 [Cyt5]	CP001287	Cyanothece sp. PCC 8801, complete genome.
	CP001288	Cyanothece sp. PCC 8801 plasmid pP880101, complete sequence.
	CP001289	Cyanothece sp. PCC 8801 plasmid pP880102, complete sequence.
	CP001290	Cyanothece sp. PCC 8801 plasmid pP880103, complete sequence.
Cyanothece sp. PCC 8802 [Cyt6]	CP001701	Cyanothece sp. PCC 8802, complete genome.
	CP001702	Cyanothece sp. PCC 8802 plasmid pP880201, complete sequence.
	CP001703	Cyanothece sp. PCC 8802 plasmid pP880202, complete sequence.
	CP001704	Cyanothece sp. PCC 8802 plasmid pP880203, complete sequence.
	CP001705	Cyanothece sp. PCC 8802 plasmid pP880204, complete sequence.
Cylindrospermum stagnale PCC 7417 [Cyli]	CP003642	Cylindrospermum stagnale PCC 7417, complete genome.
	CP003643	Cylindrospermum stagnale PCC 7417 plasmid pCYLST.01, partial sequence.
	CP003644	Cylindrospermum stagnale PCC 7417 plasmid pCYLST.02, complete sequence.
	CP003645	Cylindrospermum stagnale PCC 7417 plasmid pCYLST.03, complete sequence.
Dactylococcopsis salina PCC 8305 [Dact]	CP003944	Dactylococcopsis salina PCC 8305, complete genome.
Escherichia coli O111:H- str. 11128 [Ecol]	AP010960	Escherichia coli O111:H- str. 11128 DNA, complete genome.
	AP010961	Escherichia coli O111:H- str. 11128 plasmid pO111_1 DNA, complete sequence.
	AP010962	Escherichia coli O111:H- str. 11128 plasmid pO111_2 DNA, complete sequence.
	AP010963	Escherichia coli O111:H- str. 11128 plasmid pO111_3 DNA, complete sequence.
	AP010964	Escherichia coli O111:H- str. 11128 plasmid pO111_4 DNA, complete sequence.
	AP010965	Escherichia coli O111:H- str. 11128 plasmid pO111_5 DNA, complete sequence.
Geitlerinema sp. PCC 7407 [Geit]	CP003591	Geitlerinema sp. PCC 7407, complete genome.
Gloeobacter kilaueensis JS1 [Glo1]	CP003587	Gloeobacter kilaueensis JS1, complete genome.
Gloeobacter violaceus PCC 7421 [Glo2]	BA000045	Gloeobacter violaceus PCC 7421 DNA, complete genome.
Gloeocapsa sp. PCC 7428 [Glo3]	CP003646	Gloeocapsa sp. PCC 7428, complete genome.
	CP003647	Gloeocapsa sp. PCC 7428 plasmid pGLO7428.01, complete sequence.
	CP003648	Gloeocapsa sp. PCC 7428 plasmid pGLO7428.02, complete sequence.
	CP003649	Gloeocapsa sp. PCC 7428 plasmid pGLO7428.03, complete sequence.
	CP003650	Gloeocapsa sp. PCC 7428 plasmid pGLO7428.04, complete sequence.
Halothece sp. PCC 7418 (Aphanothece halophytica 7418) [Halo]	CP003945	Halothece sp. PCC 7418, complete genome.
Leptolyngbya sp. PCC 7376 [Lept]	CP003946	Leptolyngbya sp. PCC 7376, complete genome.

Microcoleus sp. PCC 7113 [Mic1]	CP003630	Microcoleus sp. PCC 7113, complete genome.
	CP003631	Microcoleus sp. PCC 7113 plasmid pMIC7113.01, complete sequence.
	CP003632	Microcoleus sp. PCC 7113 plasmid pMIC7113.02, complete sequence.
	CP003633	Microcoleus sp. PCC 7113 plasmid pMIC7113.03, complete sequence.
	CP003634	Microcoleus sp. PCC 7113 plasmid pMIC7113.04, complete sequence.
	CP003635	Microcoleus sp. PCC 7113 plasmid pMIC7113.05, complete sequence.
	CP003636	Microcoleus sp. PCC 7113 plasmid pMIC7113.06, complete sequence.
	CP003637	Microcoleus sp. PCC 7113 plasmid pMIC7113.07, complete sequence.
	CP003638	Microcoleus sp. PCC 7113 plasmid pMIC7113.08, complete sequence.
Microcystis aeruginosa NIES-843 [Mic2]	AP009552	Microcystis aeruginosa NIES-843 DNA, complete genome.
Nodularia spumigena CCY9414 [Nodu]	CP007203	Nodularia spumigena CCY9414 genome.
Nostoc azollae 0708 [Nos1]	CP002059	'Nostoc azollae' 0708, complete genome.
	CP002060	'Nostoc azollae' 0708 plasmid pAzo01, complete sequence.
	CP002061	'Nostoc azollae' 0708 plasmid pAzo02, complete sequence.
Nostoc punctiforme PCC 73102 (Nostoc punctiforme ATCC 29133) [Nos2]	CP001037	Nostoc punctiforme PCC 73102, complete genome.
	CP001038	Nostoc punctiforme PCC 73102 plasmid pNPUN01, complete sequence.
	CP001039	Nostoc punctiforme PCC 73102 plasmid pNPUN02, complete sequence.
	CP001040	Nostoc punctiforme PCC 73102 plasmid pNPUN03, complete sequence.
	CP001041	Nostoc punctiforme PCC 73102 plasmid pNPUN04, complete sequence.
	CP001042	Nostoc punctiforme PCC 73102 plasmid pNPUN05, complete sequence.
Nostoc sp. PCC 7107 [Nos3]	CP003548	Nostoc sp. PCC 7107, complete genome.
Nostoc sp. PCC 7120 (Anabaena sp. PCC 7120) [Nos4]	AP003602	Nostoc sp. PCC 7120 plasmid pCC7120beta DNA, complete sequence.
	AP003603	Nostoc sp. PCC 7120 plasmid pCC7120gamma DNA, complete sequence.
	AP003604	Nostoc sp. PCC 7120 plasmid pCC7120delta DNA, complete sequence.
	AP003605	Nostoc sp. PCC 7120 plasmid pCC7120epsilon DNA, complete sequence.
	AP003606	Nostoc sp. PCC 7120 plasmid pCC7120zeta DNA, complete sequence.
	BA000019	Nostoc sp. PCC 7120 DNA, complete genome.
	BA000020	Nostoc sp. PCC 7120 plasmid pCC7120alpha DNA, complete genome.
Nostoc sp. PCC 7524 (Nostoc sp. ATCC 29411) [Nos5]	CP003552	Nostoc sp. PCC 7524, complete genome.
	CP003553	Nostoc sp. PCC 7524 plasmid pNOS7524.01, complete sequence.
	CP003554	Nostoc sp. PCC 7524 plasmid pNOS7524.02, complete sequence.
Oscillatoria acuminata PCC 6304 [Osc1]	CP003607	Oscillatoria acuminata PCC 6304, complete genome.
	CP003608	Oscillatoria acuminata PCC 6304 plasmid pOSCIL6304.01, complete sequence.
	CP003609	Oscillatoria acuminata PCC 6304 plasmid pOSCIL6304.02, complete sequence.
Oscillatoria nigro-viridis PCC 7112 [Osc2]	CP003614	Oscillatoria nigro-viridis PCC 7112, complete genome.
	CP003615	Oscillatoria nigro-viridis PCC 7112 plasmid pOSC7112.01, complete sequence.
	CP003616	Oscillatoria nigro-viridis PCC 7112 plasmid pOSC7112.02, complete sequence.
	CP003617	Oscillatoria nigro-viridis PCC 7112 plasmid pOSC7112.03, complete sequence.
	CP003618	Oscillatoria nigro-viridis PCC 7112 plasmid pOSC7112.04, complete sequence.
	CP003619	Oscillatoria nigro-viridis PCC 7112 plasmid pOSC7112.05, complete sequence.
Pleurocapsa sp. PCC 7327 [Pleu]	CP003590	Pleurocapsa sp. PCC 7327, complete genome.
Prochlorococcus marinus str. AS9601 [Pr01]	CP000551	Prochlorococcus marinus str. AS9601, complete genome.
Prochlorococcus marinus str. MIT 9211 [Pr02]	CP000878	Prochlorococcus marinus str. MIT 9211, complete genome.
Prochlorococcus marinus str. MIT 9215 [Pr03]	CP000825	Prochlorococcus marinus str. MIT 9215, complete genome.
Prochlorococcus marinus str. MIT 9301 [Pr04]	CP000576	Prochlorococcus marinus str. MIT 9301, complete genome.
Prochlorococcus marinus str. MIT 9303 [Pr05]	CP000554	Prochlorococcus marinus str. MIT 9303, complete genome.
Prochlorococcus marinus str. MIT 9312 [Pr06]	CP000111	Prochlorococcus marinus str. MIT 9312, complete genome.
Prochlorococcus marinus str. MIT 9313 [Pr07]	BX548175	Prochlorococcus marinus MIT9313 complete genome.
Prochlorococcus marinus str. MIT 9515 [Pr08]	CP000552	Prochlorococcus marinus str. MIT 9515, complete genome.
Prochlorococcus marinus str. NATL1A [Pr09]	CP000553	Prochlorococcus marinus str. NATL1A, complete genome.
Prochlorococcus marinus str. NATL2A [Pr10]	CP000095	Prochlorococcus marinus str. NATL2A, complete genome.
Prochlorococcus marinus subsp. marinus str. CCMP1375 (Prochlorococcus marinus SS120) [Pr11]	AE017126	Prochlorococcus marinus subsp. marinus str. CCMP1375 complete genome.
Prochlorococcus marinus subsp. pastoris str. CCMP1986 (Prochlorococcus marinus MED4) [Pr12]	BX548174	Prochlorococcus marinus MED4 complete genome.
Prochlorococcus sp. MIT 0604 [Pr13]	CP007753	Prochlorococcus sp. MIT 0604, complete genome.
Prochlorococcus sp. MIT 0801 [Pr14]	CP007754	Prochlorococcus sp. MIT 0801, complete genome.

Pseudanabaena sp. PCC 7367<br>[Pseu]	CP003592<br>CP003593	Pseudanabaena sp. PCC 7367, complete genome.<br>Pseudanabaena sp. PCC 7367 plasmid pPSE7367.01, complete sequence.
Rivularia sp. PCC 7116<br>[Rivu]	CP003549<br>CP003550<br>CP003551	Rivularia sp. PCC 7116, complete genome.<br>Rivularia sp. PCC 7116 plasmid pRIV7116.01, complete sequence.<br>Rivularia sp. PCC 7116 plasmid pRIV7116.02, complete sequence.
Stanieria cyanosphaera PCC 7437<br>[Stan]	CP003653<br>CP003654<br>CP003655<br>CP003656<br>CP003657<br>CP003658	Stanieria cyanosphaera PCC 7437, complete genome.<br>Stanieria cyanosphaera PCC 7437 plasmid pSTA7437.01, complete sequence.<br>Stanieria cyanosphaera PCC 7437 plasmid pSTA7437.02, complete sequence.<br>Stanieria cyanosphaera PCC 7437 plasmid pSTA7437.03, complete sequence.<br>Stanieria cyanosphaera PCC 7437 plasmid pSTA7437.04, complete sequence.<br>Stanieria cyanosphaera PCC 7437 plasmid pSTA7437.05, complete sequence.
Synechococcus elongatus PCC 6301<br>(Synechococcus leopoliensis SAG 1402-1)<br>[Sy01]	AP008231	Synechococcus elongatus PCC 6301 DNA, complete genome.
Synechococcus elongatus PCC 7942<br>[Sy02]	CP000100<br>CP000101	Synechococcus elongatus PCC 7942, complete genome.<br>Synechococcus elongatus PCC 7942 plasmid 1, complete sequence.
Synechococcus sp. CC9311 [Sy03]	CP000435	Synechococcus sp. CC9311, complete genome.
Synechococcus sp. CC9605 [Sy04]	CP000110	Synechococcus sp. CC9605, complete genome.
Synechococcus sp. CC9902 [Sy05]	CP000097	Synechococcus sp. CC9902, complete genome.
Synechococcus sp. JA-2-3Ba(2-13) [Sy06]	CP000240	Synechococcus sp. JA-2-3B'a(2-13), complete genome.
Synechococcus sp. JA-3-3Ab [Sy07]	CP000239	Synechococcus sp. JA-3-3Ab, complete genome.
Synechococcus sp. KORDI-100 [Sy08]	CP006269	Synechococcus sp. KORDI-100, complete genome.
Synechococcus sp. KORDI-49 [Sy09]	CP006270	Synechococcus sp. KORDI-49, complete genome.
Synechococcus sp. KORDI-52 [Sy10]	CP006271	Synechococcus sp. KORDI-52, complete genome.
Synechococcus sp. PCC 6312<br>[Sy11]	CP003558<br>CP003559	Synechococcus sp. PCC 6312, complete genome.<br>Synechococcus sp. PCC 6312 plasmid pSYN6312.01, complete sequence.
Synechococcus sp. PCC 7002<br>[Sy12]	CP000951<br>CP000952<br>CP000953<br>CP000954<br>CP000955<br>CP000956<br>CP000957	Synechococcus sp. PCC 7002, complete genome.<br>Synechococcus sp. PCC 7002 plasmid pAQ1, complete sequence.<br>Synechococcus sp. PCC 7002 plasmid pAQ3, complete sequence.<br>Synechococcus sp. PCC 7002 plasmid pAQ4, complete sequence.<br>Synechococcus sp. PCC 7002 plasmid pAQ5, complete sequence.<br>Synechococcus sp. PCC 7002 plasmid pAQ6, complete sequence.<br>Synechococcus sp. PCC 7002 plasmid pAQ7, complete sequence.
Synechococcus sp. PCC 7502<br>[Sy13]	CP003594<br>CP003595<br>CP003596	Synechococcus sp. PCC 7502, complete genome.<br>Synechococcus sp. PCC 7502 plasmid pSYN7502.01, complete sequence.<br>Synechococcus sp. PCC 7502 plasmid pSYN7502.02, complete sequence.
Synechococcus sp. RCC307 [Sy14]	CT978603	Synechococcus sp. RCC307 genomic DNA sequence.
Synechococcus sp. WH 7803 [Sy15]	CT971583	Synechococcus WH7803 complete genome sequence.
Synechococcus sp. WH 8109 [Sy16]	CP006882	Synechococcus sp. WH 8109, complete genome.
Synechocystis sp. PCC 6803<br>[Sy17]	CP003265<br>CP003266<br>CP003267<br>CP003268<br>CP003269<br>CP003270<br>CP003271<br>CP003272	Synechocystis sp. PCC 6803, complete genome.<br>Synechocystis sp. PCC 6803 plasmid pSYSM_M, complete sequence.<br>Synechocystis sp. PCC 6803 plasmid pSYSA_M, complete sequence.<br>Synechocystis sp. PCC 6803 plasmid pSYSG_M, complete sequence.<br>Synechocystis sp. PCC 6803 plasmid pSYSX_M, complete sequence.<br>Synechocystis sp. PCC 6803 plasmid pCA2.4_M, complete sequence.<br>Synechocystis sp. PCC 6803 plasmid pCB2.4_M, complete sequence.<br>Synechocystis sp. PCC 6803 plasmid pCC5.2_M, complete sequence.
Thermosynechococcus elongatus BP-1 [The1]	BA000039	Thermosynechococcus elongatus BP-1 DNA, complete genome.
Thermosynechococcus sp. NK55 [The2]	CP006735	Thermosynechococcus sp. NK55 genome.
Trichodesmium erythraeum IMS101 [Tric]	CP000393	Trichodesmium erythraeum IMS101, complete genome.
cyanobacterium UCYN-A [UCYN]	CP001842	Cyanobacterium UCYN-A, complete genome.

Supporting Table S3: List of chromosomes and plasmids. This table enlists all 78 chromosome and 136 plasmids considered in this study. The GenBank accession ID links every sequence to the according entry in the GenBank database of the National Center for Biotechnology Information (NCBI) [http://www.ncbi.nlm.nih.gov/genbank/].
