# Supplementary material for: Modules of co-occurrence in the cyanobacterial pan-genome reveal functional associations between groups of ortholog genes
Source: PLoS Genet. 2018 Mar 9;14(3):e1007239. doi: 10.1371/journal.pgen.1007239 (PMC5862535; doi:10.1371/journal.pgen.1007239)
Supplement: S4 Table — In this table, we provide genomic and growth information for each strain including natural habitat, morphology (sections I-V, according to [61]), number of chromosomes & plasmids, number of ORFs, genome size (in megabase pairs), G+C content (in percent), fraction of DNA in ORFs (in percent), number of CLOGs, number of core CLOGs, number of shared CLOGs, number of unique CLOGs, and number of CLOGs with assigned metabolic function. We also extracted from literature the strains’ ability to fixate atmospheric nitrogen. Literature data disagreeing with the findings in our study (strain has no orthologs in module 9, composed of CLOGs mostly associated to nitrogenase) is marked with an asterisk. The last column lists various information concerning habitat, metabolism, symbiosis, and particular features of the strains. Organisms of the genus Prochlorococcus are annotated with the water depth at which the according strain was found, and their adaptation to high light (HL) or low light (LL). If not noted otherwise, data regarding the structural section was extracted from [4], while information regarding habitat, nitrogen fixation, and general properties was extracted from [62]. (PDF) [file pgen.1007239.s005.pdf]

Name	Habitat[5]	Section[12]	Chromosomes (Plasmids)	ORFs	genome size (Mb)	G+C content (%)	DNA coding (%)	CLOGs	core CLOGs	shared CLOGs	unique CLOGs	metabolic CLOGs	nitrogen fixation[5]	adaptation[5]
Acaryochloris marina MBIC11017	marine	I	1 (9)	8383	8.36	46.96	82.46	7662	620	4120	2922	3074	no	
Anabaena cylindrica PCC 7122	fresh water	IV	1 (6)	5838	7.06	38.79	79.98	5279	620	4234	425	1324	yes	Motile
Anabaena sp. 90	fresh water	IV[1]	2 (3)	4511	5.31	38.1	79.78	4161	620	3104	437	1177	yes	Motile
Anabaena variabilis ATCC 29413 *(Anabaena flos-aquae* UTEX 1444)	fresh water, soil	IV	2 (3)	5706	7.11	41.41	82.05	5070	620	4227	223	1271	yes	Motile
Arthrospira platensis NIES-39	fresh water	III	1 (0)	6630	6.79	44.27	81.22	6239	620	3234	2385	2412	no	
Calothrix sp. 336/3	fresh water	IV[2]	4 (0)	4834	6.42	41.11	71.41	4385	620	3436	329	1238	no*	h2-producing[2]
Calothrix sp. PCC 6303	fresh water	IV	1 (3)	5535	6.96	39.8	79	5001	620	3898	483	1318	yes	Photoheterotroph
Calothrix sp. PCC 7507	fresh water	IV	1 (0)	5950	7.02	42.25	79.31	5326	620	4210	496	1416	yes	
Chamaesiphon minutus PCC 6605	fresh water	I	1 (2)	5945	6.76	45.67	79.68	5436	620	3616	1200	1711	no	
Chroococcidiopsis thermalis PCC 7203	terrestrial, soil	II	1 (2)	5752	6.69	44.47	82.66	5077	620	3892	565	1473	yes	nonmotile, aerobe, heterotroph
Crinalium epipsammum PCC 9333	terrestrial[3]	III	1 (8)	4752	5.62	40.16	81.04	4383	620	3234	529	1323	no	sand dunes, drought tolerant
Cyanobacterium aponinum PCC 10605	fresh water	I	1 (1)	3431	4.18	34.93	80.09	3208	620	2360	228	1023	yes*	
Cyanobacterium stanieri PCC 7202	thermophilic, alkaline	I	1 (0)	2837	3.16	38.66	85.84	2661	620	1913	128	895	no	
Cyanobium gracile PCC 6307	fresh water	I	1 (0)	3280	3.34	68.71	89.28	3087	620	1984	483	1153	no	nonmotile, aerobe
Cyanothece sp. ATCC 51142	marine, intertidal	I	2 (4)	5304	5.46	37.94	86.31	4842	620	3463	759	1479	yes	nonmotile, photoheterotroph
Cyanothece sp. PCC 7424	fresh water, terrestrial, rice field	I	1 (6)	5710	6.55	38.51	81.1	5159	620	4048	491	1358	yes	nonmotile, photoheterotroph, anaerobe,
Cyanothece sp. PCC 7425	fresh water, terrestrial, rice field	I	1 (3)	5327	5.79	50.65	85.04	4845	620	3475	750	1483	yes	nonmotile, photoheterotroph, anaerobe,
Cyanothece sp. PCC 7822	fresh water, terrestrial, rice field	I	1 (6)	6642	7.84	39.9	82.59	6007	620	4517	870	1570	yes	nonmotile, aerobe,
Cyanothece sp. PCC 8801	fresh water, terrestrial, rice field	I	1 (3)	4367	4.79	39.76	84.54	4026	620	3334	72	1046	yes	nonmotile, aerobe,
Cyanothece sp. PCC 8802	fresh water, terrestrial, rice field	I	1 (4)	4444	4.8	39.82	84.74	4100	620	3352	128	1085	yes	nonmotile, aerobe,
Cylindrospermum stagnale PCC 7417	terrestrial, soil	IV[4]	1 (3)	6229	7.61	42.2	79.71	5659	620	4303	736	1587	yes	Photoheterotroph, aerobe
Dactylococcopsis salina PCC 8305	fresh water	I	1 (0)	3337	3.78	42.44	80.37	3129	620	2243	266	998	no	

Escherichia coli O111:H- str. 11128	homo sapiens	I[5]	1 (5)	5732	5.77	50.44	83.97	4953	404	1545	3004	3462	no	pathogenic,
Geitlerinema sp. PCC 7407	unknown[6]	III	1 (0)	3815	4.68	58.46	83.59	3544	620	2663	261	1131	yes*	motile,
Gloeobacter kilaueensis JS1	terrestrial[7]	I[7]	1 (0)	4507	4.72	60.54	90.33	4225	620	2899	706	1311	no	no thylakoid membrane,
Gloeobacter violaceus PCC 7421	terrestrial	I	1 (0)	4430	4.66	62	89.4	4111	620	2933	558	1150	no	no thylakoid membrane,
Gloeocapsa sp. PCC 7428	moderate thermophilic, fresh water	I	1 (4)	5011	5.88	43.36	82.6	4478	620	3505	353	1296	no	nonmotile, aerobe,
Halothece sp. PCC 7418 *(Aphanothece halophytica* 7418)	fresh water	I	1 (0)	3708	4.18	42.92	84.94	3447	620	2607	220	1020	no*	non-motile, anaerobe
Leptolyngbya sp. PCC 7376	cave, terrestrial	III	1 (0)	4228	5.13	43.87	82.67	3942	620	2725	597	1258	no	non-motile, aerobe
Microcoleus sp. PCC 7113	terrestrial, soil	III	1 (8)	6441	7.97	46.21	81.7	5691	620	4184	887	1685	yes	motile, anaerobe
Microcystis aeruginosa NIES-843	fresh water	I	1 (0)	6311	5.84	42.33	81.36	5894	620	3461	1813	2138	no	toxic, bloom forming, non-motile, aerobe
Nodularia spumigena CCY9414	surface, marine	IV	1 (0)	5295	5.46	41.23	80.36	4881	620	3273	988	1664	yes	toxic, bloom forming
Nostoc azollae 0708	fresh water, symbiotic	IV	1 (2)	3651	5.49	38.37	51.43	3469	620	2231	618	1204	yes	motile, aerobe, Symbiotic with duckweed
Nostoc punctiforme PCC 73102 (*Nostoc punctiforme* ATCC 29133)	fresh water, soil, symbiotic	IV	1 (5)	6690	9.06	41.35	77.2	5933	620	4728	585	1465	yes	motile, aerobe, Symbiotic with Macrozamia
Nostoc sp. PCC 7107	fresh water	IV	1 (0)	5237	6.33	40.36	80.9	4711	620	3787	304	1240	yes	aerobe
Nostoc sp. PCC 7120 *(Anabaena* sp. PCC 7120)	fresh water	IV	1 (6)	6132	7.21	41.27	82.14	5525	620	4437	468	1387	yes	aerobe
Nostoc sp. PCC 7524 (*Nostoc* sp. ATCC 29411)	fresh water	IV	1 (2)	5449	6.72	41.53	81.78	4910	620	3952	338	1318	yes	aerobe, moderate Thermal springs
Oscillatoria acuminata PCC 6304	terrestrial, soil	III	1 (2)	5796	7.8	47.61	79.64	5211	620	3858	733	1491	no	Aerobe
Oscillatoria nigro-viridis PCC 7112	terrestrial, soil	III	1 (5)	6360	8.27	45.78	77.39	5695	620	4114	961	1687	no	
Pleurocapsa sp. PCC 7327	fresh water, thermophilic	II	1 (0)	4268	4.99	45.19	81.06	3832	620	2861	351	1202	unknown* (yes)	
Prochlorococcus marinus str. AS9601	marine	I	1 (0)	1921	1.67	31.32	90.44	1881	620	1220	41	678	no	50m, HL
Prochlorococcus marinus str. MIT 9211	marine	I	1 (0)	1855	1.69	38.01	89.84	1811	620	1055	136	731	no	83m, LL
Prochlorococcus marinus str. MIT 9215	marine	I	1 (0)	1983	1.74	31.15	89.1	1931	620	1233	78	710	no	5m, HL
Prochlorococcus marinus str. MIT 9301	marine	I	1 (0)	1907	1.64	31.34	90.61	1868	620	1204	44	680	no	90m, HL
Prochlorococcus marinus str. MIT 9303	marine	I	1 (0)	2997	2.68	50.01	84.22	2911	620	1899	392	971	no	135m, HL
Prochlorococcus marinus str. MIT 9312	marine	I	1 (0)	1962	1.71	31.21	90.22	1919	620	1225	74	687	no	135m, LL

Prochlorococcus marinus str. MIT 9313	marine	I	1 (0)	2915	2.41	50.74	85.38	2850	620	1808	422	1015	no	135 m, low light adapted
Prochlorococcus marinus str. MIT 9515	marine	I	1 (0)	1906	1.7	30.79	88.32	1860	620	1181	59	674	no	15m, HL,
Prochlorococcus marinus str. NATL1A	marine	I	1 (0)	2193	1.86	34.98	86.6	2138	620	1454	64	692	no	30m, LL,
Prochlorococcus marinus str. NATL2A	marine	I	1 (0)	2163	1.84	35.12	87.01	2106	620	1439	47	684	no	30m, LL,
Prochlorococcus marinus subsp. marinus str. CCMP1375 (*Prochlorococcus marinus* SS120)	marine	I	1 (0)	1882	1.75	36.44	88.66	1834	620	1093	121	716	no	120m depth, very low light adapted,
Prochlorococcus marinus subsp. pastoris str. CCMP1986 (*Prochlorococcus marinus* MED4)	marine	I	1 (0)	1960	1.66	30.8	90.09	1922	620	1207	95	715	no	5m depth, High-light adapted,
Prochlorococcus sp. MIT 0604	marine	I[5]	1 (0)	2063	1.78	31.17	87.63	2005	620	1229	156	772	no	135m depth,
Prochlorococcus sp. MIT 0801	marine	I[5]	1 (0)	2287	1.93	34.91	85.74	2222	620	1390	212	794	no	40m depth, Low light adapted,
Pseudanabaena sp. PCC 7367	marine, intertidal[6]	III	1 (1)	3854	4.89	46.22	77.63	3590	620	2445	525	1170	no	
Rivularia sp. PCC 7116	marine	IV	1 (2)	6644	8.73	37.53	78.16	5900	620	4503	777	1609	unknown* (yes)	motile, heterotroph, aerobe
Stanieria cyanosphaera PCC 7437	fresh water	II	1 (5)	4781	5.54	36.22	81.91	4317	620	3275	422	1239	no	motile,
Synechococcus elongatus PCC 6301 (*Synechococcus leopoliensis* SAG 1402-1)	fresh water	I	1 (0)	2525	2.7	55.48	87.65	2406	620	1767	19	829	no	motile, facultative
Synechococcus elongatus PCC 7942	fresh water	I	1 (1)	2661	2.74	55.43	88.81	2537	620	1824	93	888	no	motile, facultative
Synechococcus sp. CC9311	marine, neritic	I	1 (0)	2892	2.61	52.45	86.53	2774	620	1668	486	1144	no	motile,
Synechococcus sp. CC9605	marine	I	1 (0)	2638	2.51	59.22	86.57	2542	620	1719	203	887	no	motile,
Synechococcus sp. CC9902	marine	I	1 (0)	2304	2.23	54.16	89.63	2218	620	1524	74	786	no	motile,
Synechococcus sp. JA-2-3Ba(2-13)	fresh water, thermophilic	I	1 (0)	2862	3.05	58.45	84.97	2743	620	1837	286	989	yes[8]	motile, facultative
Synechococcus sp. JA-3-3Ab	fresh water, thermophilic	I	1 (0)	2760	2.93	60.24	84.47	2628	620	1771	237	918	yes[8]	motile, facultative
Synechococcus sp. KORDI-100	marine, oligotroph	I[5]	1 (0)	3061	2.79	57.5	85.56	2965	620	1788	557	1185	no	aerobe
Synechococcus sp. KORDI-49	marine, mesophile	I[5]	1 (0)	2734	2.59	61.37	87.74	2644	620	1700	324	1002	no	aerobe
Synechococcus sp. KORDI-52	marine	I[5]	1 (0)	2820	2.57	59.09	84.46	2729	620	1769	340	1011	no	aerobe

Synechococcus sp. PCC 6312	fresh water	I	1 (1)	3545	3.72	48.5	84.67	3335	620	2256	459	1162	no	non-motile, aerobe
Synechococcus sp. PCC 7002	mud sample	I	1 (6)	3186	3.41	49.19	87.29	2999	620	2144	235	1028	no	motile, facultative
Synechococcus sp. PCC 7502	fresh water, wetland	I	1 (2)	3318	3.58	40.62	83.6	3123	620	2133	370	1061	no	sphagnum bog,
Synechococcus sp. RCC307	marine	I	1 (0)	2535	2.22	60.84	94.53	2443	620	1485	338	996	no	motile, facultative
Synechococcus sp. WH 7803	marine	I	1 (0)	2533	2.37	60.24	93.08	2428	620	1662	146	860	no	motile,
Synechococcus sp. WH 8109	marine	I	1 (0)	2644	2.11	60.09	87.91	2572	620	1548	404	1014	no	motile, facultative
Synechocystis sp. PCC 6803	fresh water	I	1 (4)	3564	3.95	47.37	86.64	3314	620	2397	297	1062	no	motile, facultative
Thermosynechococcus elongatus BP-1	thermophilic, fresh water	I	1 (0)	2475	2.59	53.92	89.79	2314	620	1586	108	802	no	
Thermosynechococcus sp. NK55	thermophilic[9]	I[9]	1 (0)	2233	2.52	53.81	85.07	2118	620	1476	22	750	no	
Trichodesmium erythraeum IMS101	marine, neritic	III	1 (0)	4451	7.75	34.14	59.86	4196	620	2808	768	1167	yes	motile, aerobe
cyanobacterium UCYN-A	symbiotic, marine[10]	I	1 (0)	1200	1.44	31.12	80.48	1173	476	664	33	495	yes[10]	symbiosis with *Braarudosphaera bigelowii*, no photosystem II, RuBisCO, TCA cycle, [11]

Supporting Table S4: General information for all strains analyzed in this study.
In this table we provide genomic and growth information for each strain including natural habitat, structural section (I-V, according to [6]), number of chromosomes&plasmids, number of ORFs, genome size (in megabase pairs), G+C content (in percent), fraction of DNA in ORFs (in percent), number of CLOGs, number of core CLOGs, number of shared CLOGs, number of unique CLOGs, and number of CLOGs with assigned metabolic function. We also extracted from literature whether the strains can fix nitrogen. Literature data disagreeing with the findings in our study is marked with an asterisk. The last column contains various information concerning habitat, metabolism, symbiosis, and particular features of the strains. If not noted otherwise, data regarding the structural section was extracted from [12], while information regarding habitat, nitrogen fixation, and general properties was extracted from [5].

References:

[1] Hao Wang et al. *Genome-derived insights into the biology of the hepatotoxic bloom-forming cyanobacterium Anabaena sp. strain 90.* BMC Genomics 2012, 13:613
[2] Janne Isojärvi et al. *Draft Genome Sequence of Calothrix Strain 336/3, a Novel H2-Producing Cyanobacterium Isolated from a Finnish Lake.* Genome Announcement 2015, vol. 3
[3] Ben de Winder et al. *Crinalium epipsammum sp. nov.: a filamentous cyanobacterium with trichomes composed of elliptical cells and containing poly-p-(1,4) glucan (cellulose).* Microbiology 1990, 136
[4] Joseph Seckbach (Ed.) *Enigmatic Microorganisms and Life in Extreme Environments.* Springer Science, vol. 1
[5] Victor M Markowitz et al. *IMG 4 version of the integrated microbial genomes comparative analysis system.* Nucleic acids research 2013
[6] Rosmarie Rippka et al. *Generic Assignments, Strain Histories and Properties of Pure Cultures of Cyanobacteria.* Microbiology 1979, 111
[7] Jimmy HW Saw et al. *Cultivation and Complete Genome Sequencing of Gloeobacter kilaueensis sp. nov., from a Lava Cave in K_lauea Caldera, Hawai'i.* 2013, e76376
[8] Anindita Bandyopadhyay et al. *Novel Metabolic Attributes of the Genus Cyanothece, Comprising a Group of Unicellular Nitrogen-Fixing Cyanobacteria.* mBio 2011, e00214
[9] Sergey Stolyar et al. *Genome Sequence of the Thermophilic Cyanobacterium Thermosynechococcus sp. Strain NK55a.* Genome Announcement 2014, vol. 2
[10] Anne W Thompson et al. *Unicellular Cyanobacterium Symbiotic with a Single-Celled Eukaryotic Alga.* Science 2012, 337
[11] Kyoko Hagino et al. *Discovery of an Endosymbiotic Nitrogen-Fixing Cyanobacterium UCYN-A in Braarudosphaera bigelowii (Prymnesiophyceae).* PLOSone 2013, e81749
[12] Patrick M Shih et al. *Improving the coverage of the cyanobacterial phylum using diversity-driven genome sequencing.* PNAS 2013, 110(3)
